# Weighted power Maxwell distribution: Statistical inference and COVID-19 applications

**DOI:** 10.1371/journal.pone.0278659

**Published:** 2023-01-03

**Authors:** Muqrin A. Almuqrin, Salemah A. Almutlak, Ahmed M. Gemeay, Ehab M. Almetwally, Kadir Karakaya, Nicholas Makumi, Eslam Hussam, Ramy Aldallal

**Affiliations:** 1 Department of Mathematics, Faculty of Science in Zulfi, Majmaah University, Al Majma’ah, Kingdom of Saudi Arabia; 2 Department of Basic Sciences, College of Science and Theoretical Studies, Saudi Electronic University, Riyadh, Saudi Arabia; 3 Department of Mathematics, Faculty of Science, Tanta University, Tanta, Egypt; 4 Faculty of Business Administration, Delta University of Science and Technology, Gamasa, Egypt; 5 The Scientific Association for Studies and Applied Research, Al Manzalah, Egypt; 6 Department of Statistics, Faculty of Sciences, Selçuk University, Konya, Turkey; 7 Pan African University, Institute for Basic Sciences, Technology and Innovation (PAUSTI), Nairobi, Kenya; 8 Department of Mathematics, Faculty of Science, Helwan University, Cairo, Egypt; 9 Department of Accounting, College of Business Administration in Hawtat Bani Tamim, Prince Sattam bin Abdulaziz University, Al-Kharj, Saudi Arabia; Amity University - Lucknow Campus, INDIA

## Abstract

During the course of this research, we came up with a brand new distribution that is superior; we then presented and analysed the mathematical properties of this distribution; finally, we assessed its fuzzy reliability function. Because the novel distribution provides a number of advantages, like the reality that its cumulative distribution function and probability density function both have a closed form, it is very useful in a wide range of disciplines that are related to data science. One of these fields is machine learning, which is a sub field of data science. We used both traditional methods and Bayesian methodologies in order to generate a large number of different estimates. A test setup might have been carried out to assess the effectiveness of both the classical and the Bayesian estimators. At last, three different sets of Covid-19 death analysis were done so that the effectiveness of the new model could be demonstrated.

## 1 Introduction

Covid-19 viruses, which have received a lot of attention over the last two years, are only one example of the massive waves of viruses we confront today. To play our part as statisticians in understanding and modelling the Covid-19 infections, It was necessary for us to devise a statistical model that could be used to fit and model the Covid-19 infections, despite the fact that these infections could be either continuous or discrete random variables. For the purpose of this study, we exerted a considerable amount of effort to select a model that offers a more satisfactory fit for the Covid-19 infections that were observed in a number of different countries. We compromised by introducing a new superior distribution as a blend of existing distributions in order to remedy the deficiencies of the standard distribution. In other words, we blended existing distributions together to create the new distribution. For more information, see [1–9]. One of the primary objectives of statistics is to develop applicable statistical models for occurrences in the real world that can be modelled using already existing probability distributions. This is one of the more important goals of statistics. In situations where the probabilities are being used to represent an event in life that could be harmful or unpredictable.

Multiple probability distributions have been developed as a result of the complexity and difficulty of simulating real-life occurrences using standard distributions. This difficulty is a direct result of the fact that standard distributions are used. There are many different applications for these distributions to be used in. There are times when the probability distributions that are already known and easily accessible are unable to accurately reflect and describe the data for particular natural occurrences. This is due to the fact that these distributions are still in the process of developing. The generalised probability distributions end up being modified and expanded as a direct consequence of the changes and expansions brought about by this research. These modifications and expansions were brought about by this research.

The accuracy with which well-known probability distributions portray the tail shape of a distribution was improved by adding a few new or additional parameters to the models. This also improved the models’ applicability to the data of real occurrences.

To show the dispersion of particles in thermal equilibrium, Maxwell (M) [10] developed a mathematical version of the Maxwell distribution. It has uni-modal and leptokurtic curves which makes it has the fact that not all molecules travel at the same speed. The Maxwell model, which has an impact on kinetic energy, explains many fundamental features of gases. It’s also known as the momenta model, velocity model, degree of momenta model, and particle energy model, and it’s used in astronomy, chemistry, and engineering. It was used as a lifetime distribution for the first time in [11], and Bayesian estimation for its parameter was provided. Chaturvedi and Rani [12] added new parameters to M model and derived Bayesian and non-Bayesian estimators. In recent research, several generalizations based on the M distribution have been presented and statistically verified. Its cumulative-distribution-function (CDF), and probability-density-function (PDF) are defined as follows

$$\begin{array}{c} F\left(x\right)=\text{erf}(\sqrt{\alpha }x)-\frac{2\sqrt{\alpha }xe^{\alpha (-x^{2})}}{\sqrt{\pi }}, \end{array}$$ (1)

and

$$\begin{array}{c} f\left(x\right)=\frac{4\alpha^{3/2}x^{2}e^{\alpha (-x^{2})}}{\sqrt{\pi }}, \end{array}$$ (2)

where $\text{erf}\left(z\right)=\frac{2}{\sqrt{\pi }}\int_{0}^{z}e^{-t^{2}}dt$.

Weighted distribution theory offers a comprehensive solution to model formulation and data interpretation issues. Weighted distributions are commonly used in investigations including dependability, survival analysis, family data analysis, biology, ecology, and a variety of other topics. For more details about weighted distributions, see [13–16].

The following constitutes the presentation of this article: In Section 2, we introduce the proposed distribution Weighted power Maxwell, along with its *PDF*, and *CDF* functions, also the graphical plot of the *PDF* and hazard rate function (HRF) are included in this section. We presented its fuzzy reliability function in section 3. Some statistical properties for the Weighted power Maxwell distributions are established in Section 4. In Section 5, conventional estimating techniques were discussed. In section 6, we applied Bayesian estimating. The simulation investigation and its associated numerical findings were completed in Section 7. Here is where Section 8’s data analysis begins. Last but not least, Section 9 contains the final observations that were extracted from this research paper.

## 2 Weighted power Maxwell distribution

To present the concept of a weighted distribution, suppose that *X* is a non-negative random variable with its PDF *f*(*x*), then the PDF of the weight random variable *X*_*w*_ is given by

$$\begin{array}{c} f_{w}\left(x\right)=\frac{w(x)f(x)}{E(w(x))},\;\;x\geq 0, \end{array}$$ (3)

where *w*(*x*) is a non-negative weight function and *E*(*w*(*x*)) = ∫_*x*_ *w*(*x*)*f*(*x*)*dx*.

Yadav et al. [17] derived the power Maxwell (PM) distribution with PDF defined as follows

$$\begin{array}{c} g\left(x\right)=\frac{4\alpha^{3/2}\beta x^{3\beta -1}e^{-\alpha x^{2\beta }}}{\sqrt{\pi }}, \end{array}$$ (4)

and its CDF is given by

$$\begin{array}{c} G\left(x\right)=1-\frac{2\alpha^{3/2}x^{3\beta }E_{-\frac{1}{2}}(x^{2\beta }\alpha )}{\sqrt{\pi }}, \end{array}$$ (5)

where $E_{n}\left(z\right)=\int_{1}^{\infty }\frac{e^{-zt}}{t^{n}}dt$, *α* > 0 is a scale parameter and *β* > 0 is a shape parameter.

The *c*^*th*^ moments of PM distribution is given by

$$\begin{array}{c} E\left(x^{c}\right)=\int_{0}^{\infty }x^{c}g\left(x\right)dx=\frac{2\alpha^{-\frac{c}{2\beta }}\Gamma (\frac{1}{2}(\frac{c}{\beta }+3))}{\sqrt{\pi }}. \end{array}$$ (6)

Let *w*(*x*) = *x*^*c*^, by using Eqs 3, 4 and 6, we have the PDF and CDF of WPM distribution as the following

$$\begin{array}{c} f\left(x\right)=\frac{2\beta \alpha^{\frac{1}{2}(\frac{c}{\beta }+3)}x^{3\beta +c-1}e^{-\alpha x^{2\beta }}}{\Gamma [\frac{1}{2}(\frac{c}{\beta }+3)]},\;\;\alpha ,\;\beta ,\;c>0, \end{array}$$ (7)

$$\begin{array}{c} F\left(x\right)=1-\frac{\Gamma [\frac{1}{2}(\frac{c}{\beta }+3),x^{2\beta }\alpha ]}{\Gamma [\frac{1}{2}(\frac{c}{\beta }+3)]}, \end{array}$$ (8)

respectively, where $\Gamma [\frac{1}{2}(\frac{c}{\beta }+3),x^{2\beta }\alpha ]$ is upper incomplete gamma function.

**Fig 1:**
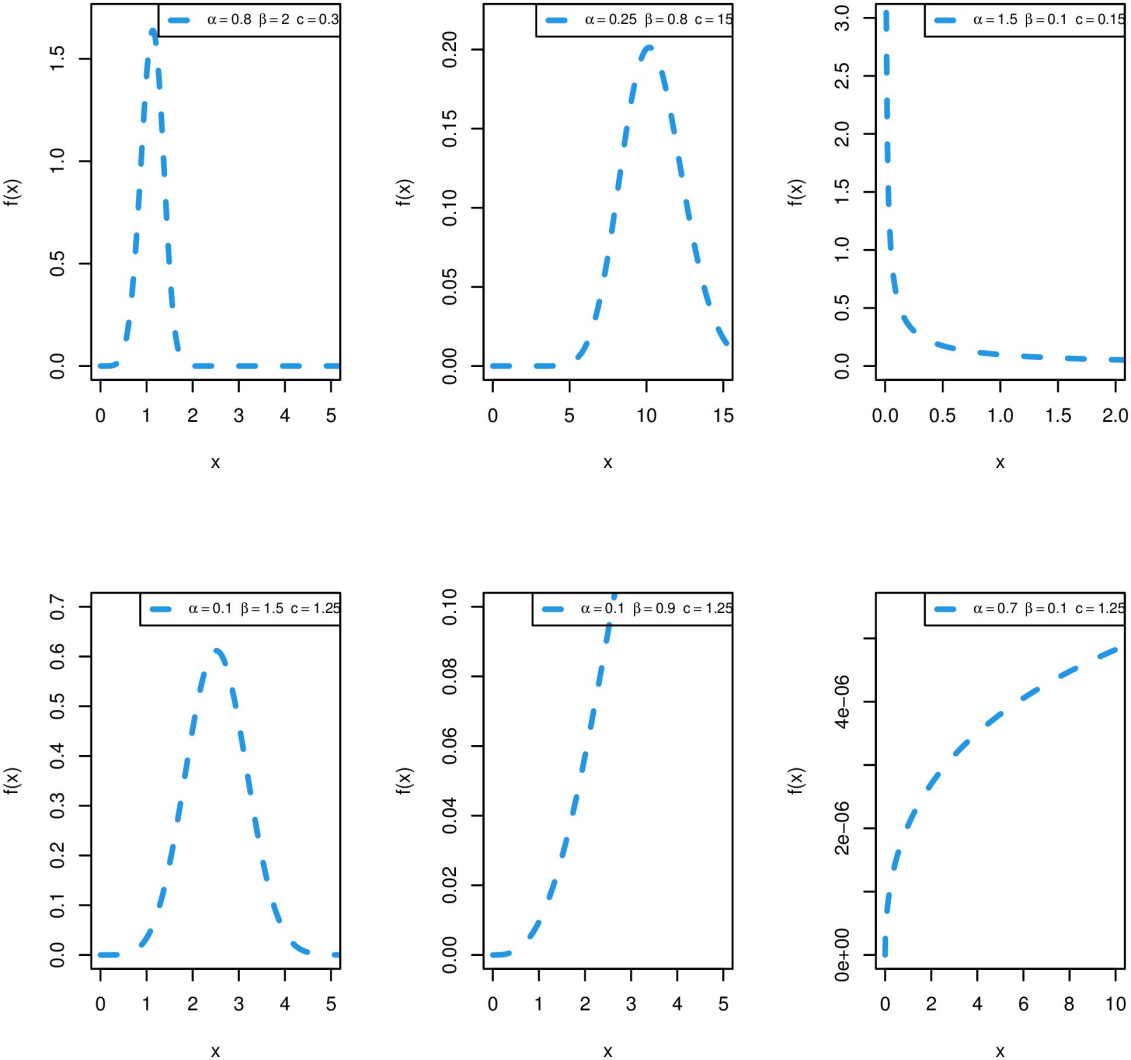
Plots of WPM PDF for different parametric values.

Plots of PDF of the WPM distribution are shown in Fig 1. The WPM distribution has a very flexible density that can be symmetric, negative skewed, positive skewed, and reversed J shaped.

**Fig 2:**
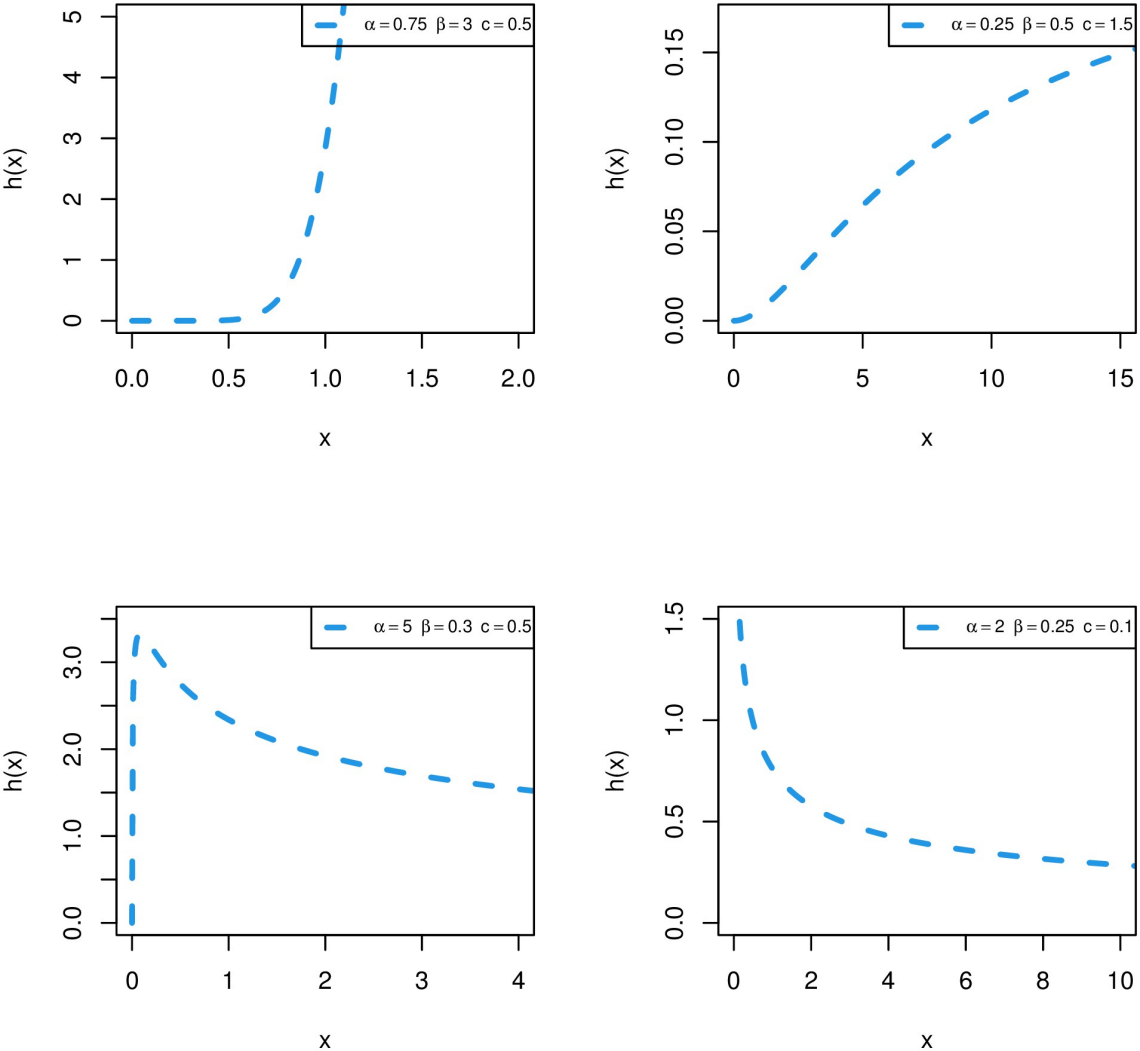
Plots of WPM HRF for different parametric values.

Plots of HRF of the WPM distribution are shown in Fig 2. The HRF of the WPM distribution can be bathtub, increasing and decreasing shaped. The shape of the HRF shows that the WPM distribution is a flexible model for data modelling.

## 3 Fuzzy and Non-Fuzzy reliability

In Non-Fuzzy reliability analysis, the survival function (SF), and HRF of WPM distribution provided by

$$\begin{array}{c} S\left(x\right)=\frac{\Gamma [\frac{1}{2}(\frac{c}{\beta }+3),x^{2\beta }\alpha ]}{\Gamma [\frac{1}{2}(\frac{c}{\beta }+3)]}, \end{array}$$ (9)

$$\begin{array}{c} h\left(x\right)=\frac{2\beta e^{-\alpha x^{2\beta }}}{xE_{-\frac{c+\beta }{2\beta }}(x^{2\beta }\alpha )}, \end{array}$$ (10)

respectively, where $E_{n}\left(z\right)=\int_{1}^{\infty }\frac{e^{-zt}}{t^{n}}dt$.

The concepts of dependability and hazard rate function are probabilities that define the life-time (a random variable) from the beginning of a failings to the point where we must modify the production procedure. The life-time is measured from the beginning of a failings to the point where we must modify the production procedure. The terms dependability function and hazard rate function are used to refer to these probabilities, respectively. As a result of the incorporation of fuzzy factors into these functions, the scope of application for dependability and hazard rate functions has been broadened, which has resulted in a wider variety of applications that can make use of these functions. The fact that the colour red was chosen for the text indicates that traditional reliability models take into account true values, which means that they take into account the lifetime probability of the system component. However, in the real world, this system lifespan accuracy is not accurate at all. This is due to the fact that the values of the system parameters obtained through experimentation, incorrect measurement are all susceptible to some degree of ambiguity. To put it another way, the accuracy of this statement is not accurate.

For the purposes of fuzzy reliability analysis, let *T* be a continuous random variable that reflects a system failure time (component). Calculating the fuzzy dependability requires only the fuzzy probability, which can be found in the formula [18].

$$\begin{array}{c} S_{F}\left(t\right)=P(T>t)=\int_{t}^{\infty }\mu \left(x\right)\;f_{WPM}\left(x\right)dx,0\leq t\leq x<\infty , \end{array}$$ (11)

where the membership function *μ*(*x*) indicates the extent to which each component of a given universe belongs to a fuzzy set. Assume now that *μ*(*x*) is

$$\begin{array}{c} \mu \left(x\right)=\frac{x-t_{1}}{t_{2}-t_{1}},\;\;\text{where}\;\;t_{1}<x<t_{2},\;\;t_{1}\geq 0 \end{array}$$ (12)

It is possible to compute the lifespan of the fuzzy numbers function *μ*(*x*), which corresponds to a specific value, and this computation yields the result of *θ* − *Cut*, *θ* ∈ [0, 1], can be obtained as: $\mu \left(x\right)=\theta \to \;\frac{x-t_{1}}{t_{2}-t_{1}}=\theta ,\;$ then

$$\begin{array}{c} x\left(\theta \right)=t_{1}+\theta \left(t_{2}-t_{1}\right),\;\;\text{where}\;\;0<\theta <1 \end{array}$$ (13)

As a result, the values of fuzzy reliability could be calculated for all *θ* values. The fuzzy dependability of the WPM distribution is determined by the fuzzy reliability definition. Thus, the fuzzy reliability values of the WPM distribution can be obtained for all values of *θ* as

$$\begin{array}{cc} S_{F}{\left(t\right)}_{0<\theta <1} & =\int_{t_{1}}^{x(\theta )}f\left(x\right)\;dx,\\ & =\frac{\Gamma [\frac{1}{2}(\frac{c}{\beta }+3),t_{1}^{2\beta }\alpha ]}{\Gamma [\frac{1}{2}(\frac{c}{\beta }+3)]}-\frac{\Gamma [\frac{1}{2}(\frac{c}{\beta }+3),x{\left(\theta \right)}^{2\beta }\alpha ]}{\Gamma [\frac{1}{2}(\frac{c}{\beta }+3)]}. \end{array}$$ (14)

## 4 Mathematical properties

### 4.1 Mode

By differentiating the logarithm of the PDF (7) concerning *x* and equating to zero, we get the mode of WPM distribution as the following

$$\begin{array}{c} x_{0}=2^{-\frac{1}{2\beta }}(\frac{3\beta +c-1}{\alpha \beta }{)}^{\frac{1}{2\beta }},\;\;c+3\beta >1, \end{array}$$

and if *c* + 3*β* ≤ 1, then WPM distribution has no mode.

### 4.2 Moments and moment generating function

The *r*^*th*^ moments of the WPM distribution is given by

$$\begin{array}{c} \mu_{r}^{\prime }=E\left(X^{r}\right)=\int_{0}^{\infty }x^{r}f\left(x\right)dx=\frac{\alpha^{-\frac{r}{2\beta }}\Gamma (\frac{c+r+3\beta }{2\beta })}{\Gamma [\frac{1}{2}(\frac{c}{\beta }+3)]}. \end{array}$$

**Fig 3:**
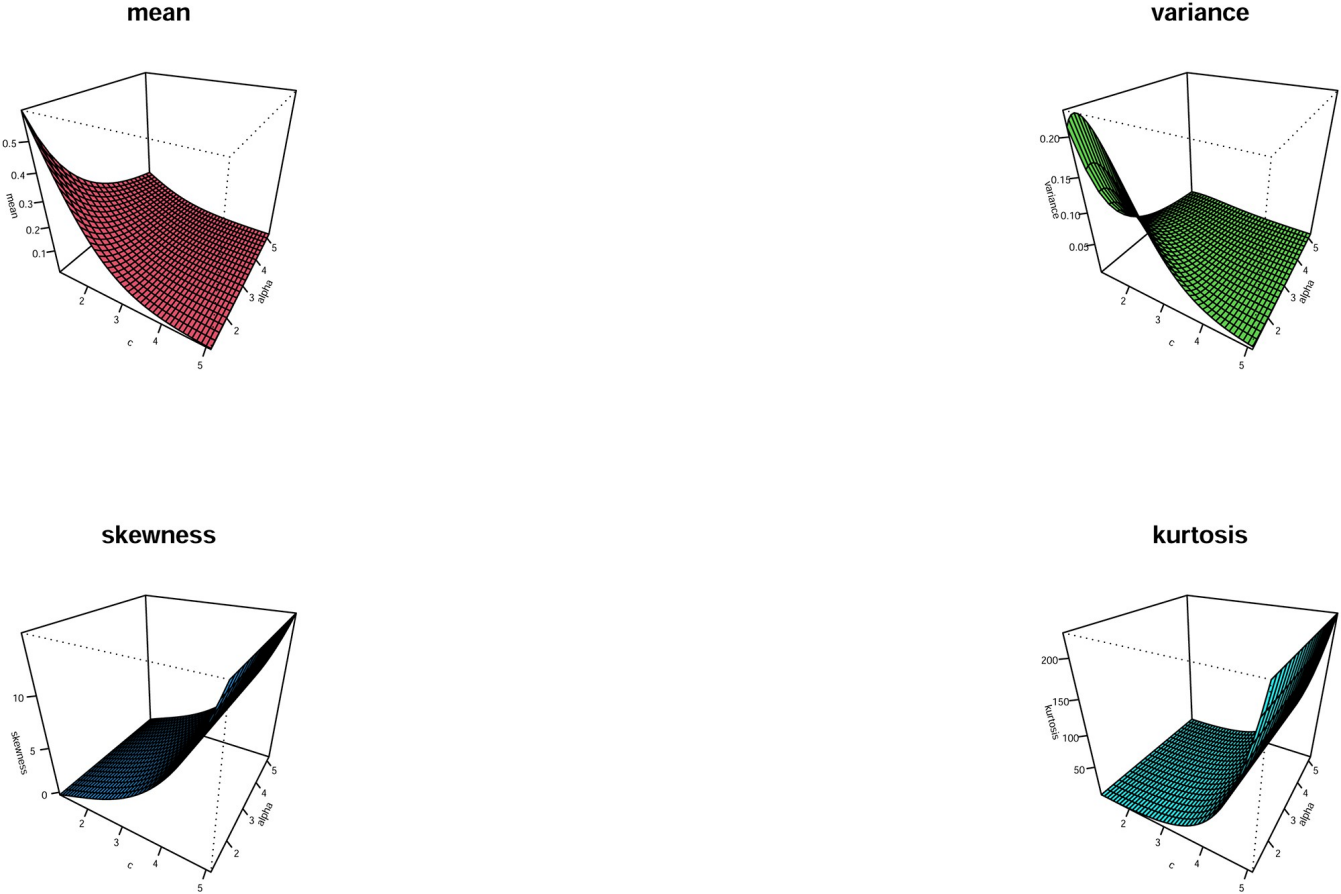
Plots of the mean, variance, skewness, and kurtosis of the HTLL model with *β* = 2.

Fig 3 shows the plots for the mean, variance, skewness, and kurtosis of the WPM model for various parametric values of *α* and *c*. It is concluded from Fig 3 that as *c* increases, the mean and variance decrease, skewness and kurtosis increase. Also, as *α* increases, the mean and variance decrease, but skewness and kurtosis keep constant.

The *r*^*th*^ incomplete moments of the WPM distribution is given by

$$\begin{array}{c} I_{r}\left(t\right)=E\left(X^{r}\right)=\int_{0}^{t}x^{r}f\left(x\right)dx=\frac{\alpha^{-\frac{r}{2\beta }}\Gamma (\frac{c+r+3\beta }{2\beta },t^{2\beta }\alpha )}{\Gamma [\frac{1}{2}(\frac{c}{\beta }+3)]}, \end{array}$$

where $\Gamma (\frac{c+r+3\beta }{2\beta },t^{2\beta }\alpha )$ is lower incomplete gamma function.

The moment generating function of the WPM distribution is given by

$$\begin{array}{c} M\left(t\right)=\sum_{k=0}^{\infty }\frac{t^{k}\alpha^{-\frac{k}{2\beta }}\Gamma (\frac{c+k+3\beta }{2\beta })}{\Gamma [\frac{1}{2}(\frac{c}{\beta }+3)]}. \end{array}$$

We can replace *t* with *it* to get the characteristics function.

### 4.3 Mean residual life and mean inactivity time

The mean residual life (MRL) of WPM distribution is defined as the following

$$\begin{array}{c} MRL=\frac{1-I_{1}\left(t\right)}{S(t)-t}=-\frac{\Gamma [\frac{1}{2}(\frac{c}{\beta }+3)]-\alpha^{-\frac{1}{2\beta }}\Gamma (\frac{c+3\beta +1}{2\beta })}{t\Gamma [\frac{1}{2}(\frac{c}{\beta }+3)]-\Gamma [\frac{1}{2}(\frac{c}{\beta }+3),t^{2\beta }\alpha ]}, \end{array}$$

where *I*_1_(*t*) is first incomplete moments and $\Gamma [\frac{1}{2}(\frac{c}{\beta }+3),t^{2\beta }\alpha ]$ is upper incomplete gamma function.

The mean inactivity time (MIT) is defined as the waiting time elapsed since the failure of an item on the condition that this failure had occurred in (0, *t*). The MIT of WPM distribution is defined as the following

$$\begin{array}{c} MIT=t-\frac{I_{1}\left(t\right)}{F(t)}=t-\frac{\alpha^{-\frac{1}{2\beta }}\Gamma (\frac{c+3\beta +1}{2\beta })}{\Gamma [\frac{1}{2}(\frac{c}{\beta }+3)]-\Gamma [\frac{1}{2}(\frac{c}{\beta }+3),t^{2\beta }\alpha ]}, \end{array}$$

where $\Gamma [\frac{1}{2}(\frac{c}{\beta }+3),t^{2\beta }\alpha ]$ is the upper incomplete gamma function.

### 4.4 Order statistics

The *i*th order statistic’s PDF and CDF for the WPM distribution are shown.

fi:n(x)=n!(i-1)!(n-i)![F(x)]i-1[1-F(x)]n-if(x)=2βn!α12(cβ+3)x3β+c-1e-αx2βΓ[12(cβ+3)]-n×Γ[12(cβ+3),t2βα]n-i{Γ[12(cβ+3)]-Γ[12(cβ+3),t2βα]}i-1Γ(i)Γ(-i+n+1),Fi:n(x)=∑r=in(rn)[F(x)]r[1-F(x)]n-r=(ni){1-Γ[12(cβ+3),x2βα]Γ[12(cβ+3)]}i{Γ[12(cβ+3),x2βα]Γ[12(cβ+3)]}n-i×2F1{1,i-n;i+1;1-Γ[12(cβ+3)]Γ[12(cβ+3),x2βα]},

where ${\;}_{2}F_{1}\{1,i-n;i+1;1-\frac{\Gamma [\frac{1}{2}(\frac{c}{\beta }+3)]}{\Gamma [\frac{1}{2}(\frac{c}{\beta }+3),x^{2\beta }\alpha ]}\}$ is a hypergeometric function and $\Gamma [\frac{1}{2}(\frac{c}{\beta }+3),t^{2\beta }\alpha ]$ is upper incomplete gamma function.

see [19] for more reading.

$$\begin{array}{c} \underset{n\to +\infty }{\text{lim}}\;P\left(W_{n}<d_{n}x\right)=1-\text{exp}\left(-x^{c+3\beta }\right),\;x>0,\;d_{n}=F^{-1}(\frac{1}{n}). \end{array}$$

$$\begin{array}{c} \underset{n\to +\infty }{\text{lim}}P(Z_{n}<b_{n}x)=\{\begin{array}{ll} \infty & ,\;0<x^{2\beta }<1,\\ e^{-x^{c+\beta }} & ,\;\;x^{2\beta }=1,\\ 0 & ,\;\;x^{2\beta }>1, \end{array} \end{array}$$

where $b_{n}=F^{-1}(1-\frac{1}{n})$.

### 4.5 Inequality curves

The Lorenz curve is the most extensively used and well-known of the inequality curves, and it has applications in a wide range of domains.

It is defined for the WPM distribution as follows:

$$\begin{array}{ccc} L(p) & = & \frac{I_{1}\left(x_{p}\right)}{\mu }=1-\frac{\Gamma (\frac{c+3\beta +1}{2\beta },\alpha x_{p}^{2\beta })}{\Gamma (\frac{c+3\beta +1}{2\beta })}, \end{array}$$

where *F*(*x*_*p*_) = *p*, *I*_1_(*t*) is first incomplete moments, *x*_*p*_ is the quantile function and $\Gamma (\frac{c+3\beta +1}{2\beta },\alpha x_{p}^{2\beta })$ upper incomplete gamma function.

Also, we can determine Bonferroni and Zenga inequality curves according to their relationship with the Lorenz curve as the following (for more details see [20])

$$\begin{array}{c} B\left(p\right)=\frac{L(p)}{p},\;Z\left(p\right)=\frac{L(p)-p}{p[1-L(p)]}. \end{array}$$

### 4.6 Entropies

This subsection contains different types of entropies for our proposed model such as Rényi, Tsallis, and Shannon entropies, for more details see [21–23]. The Rényi, *K*_*X*_(*r*), and Tsallis, *L*_*X*_(*r*) entropies of order *r*, where *r* > 0, *r* ≠ 1 of our proposed distribution are given, respectively, by

$$\begin{array}{ccc} K_{X}\left(r\right) & = & \frac{1}{1-r}\text{log}\;\int_{x=0}^{\infty }f^{S}\left(x\right)dx,\;r>0,r\neq 1\\ & = & \frac{1}{r-1}\;\text{log}\;(\frac{2^{r-1}\alpha^{\frac{1}{2}r(\frac{c}{\beta }+3)}{\left(\alpha r\right)}^{-\frac{r(3\beta +c-1)+1}{2\beta }}\{\frac{\beta }{\Gamma [\frac{1}{2}(\frac{c}{\beta }+3)]}{\}}^{r}\Gamma [\frac{r(c+3\beta -1)+1}{2\beta }]}{\beta }) \end{array}$$

and

$$\begin{array}{ccc} L_{X}\left(r\right) & = & \frac{1}{1-r}[\int_{x=0}^{\infty }f^{r}\left(x\right)dx-1]\\ & = & \frac{(\frac{2^{r-1}\alpha^{\frac{1}{2}r(\frac{c}{\beta }+3)}{\left(\alpha r\right)}^{-\frac{r(3\beta +c-1)+1}{2\beta }}\{\frac{\beta }{\Gamma [\frac{1}{2}(\frac{c}{\beta }+3)]}{\}}^{r}\Gamma [\frac{r(c+3\beta -1)+1}{2\beta }]}{\beta })-1}{1-r}. \end{array}$$

The Shannon entropy of WPM distribution is determined as follows

$$\begin{array}{ccc} H_{X}\left(1\right) & = & \underset{r⟶1}{\text{lim}}\;K_{X}\left(r\right)\\ & = & -\frac{\text{log}\left(\alpha \right)-3\beta +\beta \;\text{log}\;\left(4\right)+2\beta \;\text{log}\left(\beta \right)+\left(3\beta +c-1\right)\Phi (\frac{c+3\beta }{2\beta })-2\beta \;\text{log}(\Gamma [\frac{c+3\beta }{2\beta }])-c}{2\beta }, \end{array}$$

where $\Phi \left(z\right)=\frac{d}{dz}\text{log}\left[\Gamma \left(z\right)\right]$.

### 4.7 Stress-strength reliability

Assume that *X* and *Y* are two independent random variables drawn from the WPM distribution with parameters (*α*, *β*, *c*_1_) and (*α*, *β*, *c*_2_), respectively. Then by using CDF 8 and PDF 7, we have stress-strength reliability of WPM distribution as follows

$$\begin{array}{cc} R & =P\left(X>Y\right)=\int_{-\infty }^{\infty }F_{Y}\left(x\right)f_{X}\left(x\right)dx\\ & =\frac{2\beta \alpha^{\frac{1}{2}(\frac{c_{1}}{\beta }+3)}}{\Gamma (\frac{1}{2}(\frac{c_{1}}{\beta }+3))}\int_{0}^{\infty }(x^{3\beta +c_{1}-1}e^{\alpha (-x^{2\beta })})(1-\frac{\Gamma (\frac{1}{2}(\frac{c_{2}}{\beta }+3),x^{2\beta }\alpha )}{\Gamma (\frac{1}{2}(\frac{c_{2}}{\beta }+3))})dx\\ & =\frac{2\beta \alpha^{\frac{1}{2}(\frac{c_{1}}{\beta }+3)}}{\Gamma (\frac{1}{2}(\frac{c_{1}}{\beta }+3))\Gamma (\frac{1}{2}(\frac{c_{2}}{\beta }+3))}\int_{0}^{\infty }(x^{3\beta +c_{1}-1}e^{\alpha (-x^{2\beta })})Adx, \end{array}$$

where $A=\gamma \left(\frac{1}{2}(\frac{c_{2}}{\beta }+3),\alpha x^{2\beta }\right)=\int_{0}^{\alpha x^{2\beta }}t^{\frac{1}{2}(\frac{c_{2}}{\beta }+3)-1}e^{-t}dt$. From Wikipedia (https://en.wikipedia.org/wiki/Incomplete_gamma_function), we have

$$\begin{array}{c} \gamma \left(a,x\right)=e^{-x}x^{a}\Gamma \left(a\right)\sum_{n=0}^{\infty }\frac{x^{n}}{\Gamma (a+n+1)}, \end{array}$$

Then we have

$$\begin{array}{cc} R & =\sum_{n=0}^{\infty }\frac{2\beta \alpha^{\frac{c_{1}+c_{2}+2\beta \left(n+3\right)}{2\beta }}}{\Gamma (\frac{1}{2}(\frac{c_{1}}{\beta }+3))\Gamma (\frac{1}{2}(\frac{c_{2}}{\beta }+3)+n+1)}\int_{0}^{\infty }(x^{3\beta +c_{1}-1}e^{\alpha (-x^{2\beta })})e^{\alpha (-x^{2\beta })}x^{\beta (\frac{c_{2}}{\beta }+3)+2\beta n}dx\\ & =\sum_{n=0}^{\infty }\frac{2^{1-\frac{c_{1}+c_{2}+2\beta \left(n+4\right)}{2\beta }}\alpha^{\frac{1}{2}(\frac{c_{1}}{\beta }+3)}}{\Gamma (\frac{1}{2}(\frac{c_{1}}{\beta }+3))\Gamma (\frac{1}{2}(\frac{c_{2}}{\beta }+3)+n+1)}\Gamma (\frac{2\left(n+3\right)\beta +c_{1}+c_{2}}{2\beta }). \end{array}$$

**Fig 4:**
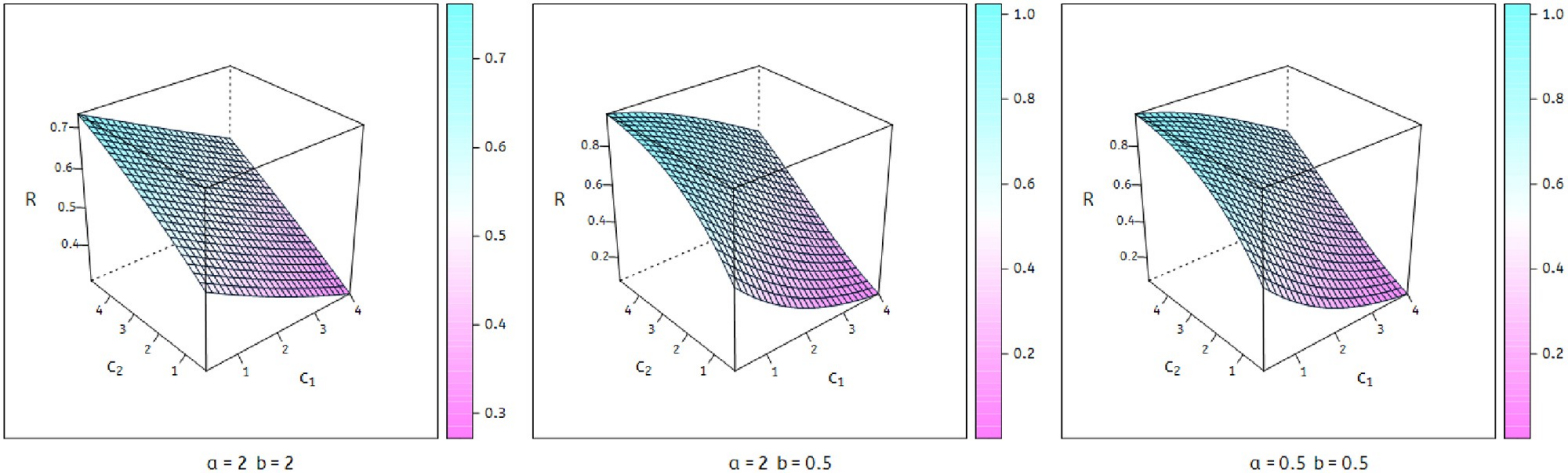
Plots of stress-strength reliability.

Fig 4 show Stress-strength reliability of WPM model has different values by using different values of parameters.

## 5 Maximum likelihood estimation

Let *x*_1_, *x*_2_, …, *x*_*n*_ be a random sample of size *n* from the PDF of the WPM model, then the likelihood function takes the form

$$\begin{array}{c} L\left(\Theta \right)=\frac{2^{n}\beta^{n}\alpha^{\frac{n}{2}(\frac{c}{\beta }+3)}e^{-\alpha \sum_{i=1}^{n}x_{i}^{2\beta }}\prod_{i=1}^{n}x_{i}^{3\beta +c-1}}{{\left\{\Gamma [\frac{1}{2}(\frac{c}{\beta }+3)]\right\}}^{n}}, \end{array}$$ (15)

where Θ is the parameter vector (*α*, *β*, *c*). Then the log-likelihood function takes the form

$$\begin{array}{c} \ell \left(\Theta \right)=n[\text{ln}\left(2\right)+\text{ln}\left(\beta \right)+\frac{1}{2}(\frac{c}{\beta }+3)\;\text{ln}\left(\alpha \right)-\text{ln}\{\Gamma [\frac{1}{2}(\frac{c}{\beta }+3)]\}]-\alpha \sum_{i=1}^{n}x_{i}^{2\beta }+(3\beta +c-1)\sum_{i=1}^{n}\text{ln}\left(x_{i}\right). \end{array}$$ (16)

We have estimators of the parameters *α*, *β* and *c* of the proposed model via the MLE by differentiating Eq (16) with regard to *α*, *β* and *c*, respectively, and equating to zero.

$$\begin{array}{c} \frac{\partial \ell (\Theta )}{\partial \alpha }=\frac{n(\frac{c}{\beta }+3)}{2\alpha }-\sum_{i=1}^{n}x_{i}^{2\beta }, \end{array}$$ (17)

$$\begin{array}{c} \frac{\partial \ell (\Theta )}{\partial \beta }=\frac{n}{\beta }-\frac{n}{2}\frac{c}{\beta^{2}}\text{ln}\left(\alpha \right)-n\frac{{\overset{´}{\Gamma }}_{\beta }[\frac{1}{2}(\frac{c}{\beta }+3)]}{\Gamma [\frac{1}{2}(\frac{c}{\beta }+3)]}-2\alpha \sum_{i=1}^{n}x_{i}^{2\beta }\;\text{ln}\left(x_{i}\right)+3\sum_{i=1}^{n}\text{ln}\left(x_{i}\right), \end{array}$$ (18)

and

$$\begin{array}{c} \frac{\partial \ell (\Theta )}{\partial c}=\frac{n}{2\beta }\text{ln}\left(\alpha \right)-n\frac{{\overset{´}{\Gamma }}_{c}[\frac{1}{2}(\frac{c}{\beta }+3)]}{\Gamma [\frac{1}{2}(\frac{c}{\beta }+3)]}+\sum_{i=1}^{n}\text{ln}\left(x_{i}\right). \end{array}$$ (19)

where ${\overset{´}{\Gamma }}_{x}\left(x\right)=\int_{0}^{\infty }t^{x-1}e^{-t}\;\text{ln}\left(t\right)dt$ then ${\overset{´}{\Gamma }}_{c}[\frac{1}{2}(\frac{c}{\beta }+3)]=\frac{\partial \Gamma [\frac{1}{2}(\frac{c}{\beta }+3)]}{\partial c}=\frac{c}{2}\int_{0}^{\infty }t^{\frac{1}{2}(\frac{c}{\beta }+3)-1}e^{-t}\;\text{ln}\left(t\right)dt$, and ${\overset{´}{\Gamma }}_{\beta }[\frac{1}{2}(\frac{c}{\beta }+3)]=\frac{\partial \Gamma [\frac{1}{2}(\frac{c}{\beta }+3)]}{\partial \beta }=\frac{-c}{2\beta^{2}}\int_{0}^{\infty }t^{\frac{1}{2}(\frac{c}{\beta }+3)-1}e^{-t}\;\text{ln}\left(t\right)dt$.

As it seems, from (17), (18) and (19), analytic solutions of MLEs of *α*, *β* and *c* are not available. Therefore, the “maxLik” package can be used numerically to perform an iterative Newton-Raphson (NR) approach in order to achieve the appropriate MLEs $\hat{\alpha }$, $\hat{\beta },$ and $\hat{c}$ for any given data sets. Once the maximum likelihood estimates of *α*, *β* and *c* calculated, the MLEs of the non-fuzzy reliability and fuzzy reliability indices *S*(*t*) (9) at any mission time *t* > 0 can be easily derived using the invariance property of MLEs $\hat{\alpha },\hat{\beta }$ and $\hat{c}$ as

$$\begin{array}{c} \hat{S}\left(t\right)=\frac{\Gamma [\frac{1}{2}(\frac{\hat{c}}{\hat{\beta }}+3),t^{2\hat{\beta }}\hat{\alpha }]}{\Gamma [\frac{1}{2}(\frac{\hat{c}}{\hat{\beta }}+3)]},\;t>0, \end{array}$$

and

$$\begin{array}{c} {\hat{S}}_{F}\left(t\right)=\frac{\Gamma [\frac{1}{2}(\frac{\hat{c}}{\hat{\beta }}+3),t_{1}^{2\hat{\beta }}\alpha ]}{\Gamma [\frac{1}{2}(\frac{\hat{c}}{\hat{\beta }}+3)]}-\frac{\Gamma [\frac{1}{2}(\frac{\hat{c}}{\hat{\beta }}+3),x{\left(\theta \right)}^{2\hat{\beta }}\hat{\alpha }]}{\Gamma [\frac{1}{2}(\frac{\hat{c}}{\hat{\beta }}+3)]},\;t>0. \end{array}$$

### 5.1 Asymptotic confidence intervals

To build the two-sided 100(1−*γ*)% ACIs for the unknown parameters *α*, *β*, and *c*, or any function of them such as *S*(*t*) and *S*_*F*_(*t*), say Θ = (*α*, *β*, *c*, *S*(*t*), *S*_*F*_(*t*)), the Fisher’s information matrix ${\text{I}}_{ij}\left(\underline{\theta }\right)=E[-(\partial^{2}\ell (\underline{\theta }|\underline{x}))/\partial {\underline{\theta }}^{2}\;],\;i,j=1,2$. Since the exact solutions of the Fisher’s expectation is tedious to obtain, hence the asymptotic variance-covariance (V-C) matrix of the MLEs $\hat{\alpha },\hat{\beta }$ and $\hat{c}$ can be obtained by inverting $\text{I}(\underline{\alpha })$ and dropping *E* with replacing *α*, *β* and *c* by their MLEs $\hat{\alpha },\hat{\beta }$ and $\hat{c}$, respectively, see [24]. Also, [25] stated that the MLE $\hat{\Theta }$ and MPSE $\hat{\Theta }$ are asymptotically analogous, in fact, $\hat{\Theta }=\hat{\Theta }+o\left(n^{-1/2}\right)$.

However, by differentiating (16) partially with respect to *α*, *β* and *c*, locally at their MLEs $\hat{\alpha },\hat{\beta }$ and $\hat{c}$, the approximate V-C matrix, ${\text{I}}^{-1}\left(\hat{\alpha },\hat{\beta }\hat{c}\right)$, is given by

$$\begin{array}{c} {\text{I}}^{-1}\left(\hat{\alpha }\hat{\beta },\hat{c}\right)=[\begin{array}{ccc} -\ell_{11} & -\ell_{12} & -\ell_{13}\\ -\ell_{21} & -\ell_{22} & -\ell_{23}\\ -\ell_{31} & -\ell_{32} & -\ell_{33} \end{array}{]}_{\left(\hat{\alpha },\hat{\beta },\hat{c}\right)}^{-1}=[\begin{array}{ccc} {\hat{\sigma }}_{\hat{\alpha }\hat{\alpha }} & {\hat{\sigma }}_{\hat{\alpha }\hat{\beta }} & {\hat{\sigma }}_{\hat{\alpha }\hat{c}}\\ {\hat{\sigma }}_{\hat{\beta }\hat{\theta }} & {\hat{\sigma }}_{\hat{\beta }\hat{\beta }} & {\hat{\sigma }}_{\hat{\beta }\hat{\beta }}\\ {\hat{\sigma }}_{\hat{c}\hat{\theta }} & {\hat{\sigma }}_{\hat{c}\hat{\beta }} & {\hat{\sigma }}_{\hat{c}\hat{c}} \end{array}]. \end{array}$$ (20)

To construct the ACIs of *S*(*t*) and *S*_*F*_(*t*), one needs to obtain the corresponding estimated variances. Practically, the most statistically efficient method used to construct confidence intervals is called the delta approach. This approach is useful and easy to utilize compared to the empirically-driven bootstrap approach due to the latter should be taken as a second-choice alternative when the Taylor series approximation is empirically incorrect, see [26]. However, based on the asymptotic normality of the MLEs of the reliability parameters of life *S*(*t*) and *S*_*F*_(*t*), we have $\hat{S}\left(t\right)∼N\left(\hat{S}\left(t\right),\sigma_{\hat{S}\left(t\right)}^{2}\right)$ and ${\hat{S}}_{F}\left(t\right)∼N\left({\hat{S}}_{F}\left(t\right),\sigma_{{\hat{S}}_{F}\left(t\right)}^{2}\right)$. According to the delta method, from (20), the ACIs for *S*(*t*) and *S*_*F*_(*t*) can be constructed using the corresponding normality, respectively as

$$\begin{array}{c} {\hat{\sigma }}_{\hat{S}\left(t\right)}^{2}=[\Delta_{S(t)}{\text{I}}^{-1}\left(\alpha ,\beta ,c\right)\Delta_{S(t)}^{T}]{|}_{\left(\hat{\theta },\hat{c}\right)},\;\text{and}\;{\hat{\sigma }}_{{\hat{S}}_{F}\left(t\right)}^{2}=[\Delta_{h(t)}{\text{I}}^{-1}\left(\alpha ,\beta ,c\right)\Delta_{S_{F}\left(t\right)}^{T}]{|}_{\left(\hat{\theta },\hat{c}\right)}, \end{array}$$

where Δ_*S*(*t*)_ and $\Delta_{S_{F}(t)}$ are the gradient of *S*(*t*) and *S*_*F*_(*t*) obtained at $\hat{\alpha },\hat{\beta }$ and $\hat{c}$ as

$$\begin{array}{c} \Delta_{S(t)}=[\frac{\partial S_{F}\left(t\right)}{\partial \alpha },\frac{\partial S_{F}\left(t\right)}{\partial \beta },\frac{\partial S_{F}\left(t\right)}{\partial c}\;],\;\text{and}\;\Delta_{S_{F}\left(t\right)}=[\frac{\partial S_{F}\left(t\right)}{\partial \alpha },\frac{\partial S_{F}\left(t\right)}{\partial \beta },\frac{\partial S_{F}\left(t\right)}{\partial c}]. \end{array}$$

Hence, using the concept of large sample theory for MLEs $\hat{\Theta }$ of Θ, the 100(1 − *γ*)% two-sided ACIs for the unknown parameter *α*, *β*, *c*, *S*(*t*) and *S*_*F*_(*t*) is given by

$$\begin{array}{c} \hat{\Theta }\mp z_{\gamma /2\;}\sqrt{{\hat{\sigma }}_{\hat{\Theta }}^{2}}, \end{array}$$

where ${\hat{\sigma }}_{\hat{\Theta }}^{2}$ is the estimated variance of $\hat{\Theta }$, and *z*_*γ*/2_ is the percentile of the standard normal distribution with upper probability (*γ*/2) − *th*.

## 6 Bayesian estimators

The Bayesian paradigm has become the most common technique in several sectors in recent years, including but not limited to different applications. Its capacity to use prior information in the analysis makes it particularly valuable in dependability studies, where one of the major obstacles is data availability. The Bayes estimates and associated credible interval of the model parameters *α*, *β*, and *c*, as well as the fuzzy and non-fuzzy reliability parameters *S*(*t*) and *S*_*F*_(*t*), are discussed in this section.

### 6.1 Prior distribution and loss function

Because the gamma prior distribution can take on a variety of shapes depending on its parameter values, using separate gamma priors is a relatively simple process that can lead to discoveries with more explicit posterior density expressions. As a result, we evaluated gamma density priors, which are more adjustable in terms of altering support for the WPM distribution parameters than other difficult prior distributions. Thus, the WPM parameters *α*, *β*, and *c* are assumed to have independent gamma PDFs as *Gamma*(*q*_1_, *w*_1_), *Gamma*(*q*_2_, *w*_2_) and *Gamma*(*q*_3_, *w*_3_), respectively. Then, the joint prior density of *α*, *β*, and *c* becomes

$$\begin{array}{c} \pi (\alpha ,\beta ,c)\propto \alpha^{q_{1}-1}\beta^{q_{2}-1}c^{q_{3}-1}e^{-(w_{1}\alpha +w_{2}\beta +w_{3}c)},\;\alpha ,\beta ,c>0, \end{array}$$ (21)

where the hyperparameters *q*_*i*_, *w*_*i*_, *i* = 1, 2, 3, are chosen to reflect prior knowledge about the unknown parameters *α*, *β* and *c*, and they assumed to be known and non-negative, we determined the hyperparameters by eliciting hyper-parameters as El-Sherpieny et al. [27].

In literature, the choice of symmetric and asymmetric loss functions are an important issue in Bayesian analysis. So in this study for estimating the considered unknown quantities, the most widely-used symmetric loss function is the SEL function, L(·), which is defined as

$$\begin{array}{c} L(\Theta ,\tilde{\Theta })={\left(\tilde{\Theta }-\Theta \right)}^{2}, \end{array}$$ (22)

where $\tilde{\Theta }$ being an estimate of Θ. Under (22), objective estimate $\tilde{\Theta }$ is given by the posterior mean of Θ. However, any other loss function can be easily incorporated.

The entropy loss function (ELF) is a good asymmetric loss function, according to Calabria and Pulcini [28]. The entropy loss function of the form is considered as

$$\begin{array}{c} L_{E}\left(\tilde{\Theta },\Theta \right)\propto (\frac{\tilde{\Theta }}{\Theta }{)}^{b}-b\;\text{ln}(\frac{\tilde{\Theta }}{\Theta })-1, \end{array}$$ (23)

whose minimum occurs at $\tilde{\Theta }=\Theta$. Then the Bayes estimator of Θ under entropy loss function is

$$\begin{array}{c} {\tilde{\Theta }}_{E}=[E_{\Theta }(\Theta^{-b}){]}^{\frac{-1}{b}}, \end{array}$$ (24)

### 6.2 Posterior analysis

The joint posterior density function, *π*_*L*_(⋅) of *α*, *β* and *c* is given by

$$\begin{array}{c} \pi_{L}(\alpha ,\beta ,c|\underline{x})=K_{1}^{-1}\pi (\alpha ,\beta ,c)L(\alpha ,\beta ,c|\underline{x}), \end{array}$$ (25)

where $K_{1}=\int_{0}^{\infty }\int_{0}^{\infty }\pi_{L}(\alpha ,\beta ,c|\underline{x})d\alpha d\beta dc$ is the normalizing constant.

Substituting (15) and (21) into (25), the joint posterior PDF of *α*, *β* and *c* becomes

$$\pi_{L}(\alpha ,\beta ,c|\underline{x})=K_{1}^{-1}\frac{2^{n}\beta^{n+q_{2}-1}c^{q_{3}-1}\alpha^{\frac{n}{2}(\frac{c}{\beta }+3)+q_{1}-1}e^{-(\beta w_{2}+cw_{3})}e^{-\alpha (w_{1}+\sum_{i=1}^{n}x_{i}^{2\beta })}\prod_{i=1}^{n}x_{i}^{3\beta +c-1}}{{\left\{\Gamma [\frac{1}{2}(\frac{c}{\beta }+3)]\right\}}^{n}}.$$ (26)

Under SELF (22) and ELF (24), the Bayesian estimator for any function of *α*, *β* and *c*, say Θ, is the posterior expectation of Θ. Therefore, it is necessary to acquire the marginal posterior distributions for each parameter of *α*, *β*, and *c* in order to develop these estimations, because (26) implicit mathematical expressions. It is quite evident that obtaining explicit forms for the marginal PDFs for each unknown parameter is not even remotely conceivable. In light of this, our intention is to use certain simulation techniques, such as the MCMC approaches, in order to generate the Bayesian estimates and the credible intervals that correspond to them.

First, from (26), we obtain the conditional posterior distributions of the supplied data for *α*, *β*, and *c* as follows:

$$\begin{array}{c} \pi_{L}(\alpha |\beta ,c,\underline{x})\propto \alpha^{\frac{n}{2}(\frac{c}{\beta }+3)+q_{1}-1}e^{-\alpha (w_{1}+\sum_{i=1}^{n}x_{i}^{2\beta })}, \end{array}$$ (27)

$$\begin{array}{c} \pi_{L}(\beta |\alpha ,c,\underline{x})\propto \frac{\beta^{n+q_{2}-1}e^{-\beta w_{2}}e^{-\alpha \sum_{i=1}^{n}x_{i}^{2\beta }}\prod_{i=1}^{n}x_{i}^{3\beta }}{{\left\{\Gamma [\frac{1}{2}(\frac{c}{\beta }+3)]\right\}}^{n}}, \end{array}$$ (28)

and

$$\begin{array}{c} \pi_{L}(c|\alpha ,\beta ,\underline{x})\propto \frac{c^{q_{3}-1}\alpha^{\frac{n}{2}\;\frac{c}{\beta }}e^{-cw_{3}}\prod_{i=1}^{n}x_{i}^{c-1}}{{\left\{\Gamma [\frac{1}{2}(\frac{c}{\beta }+3)]\right\}}^{n}}. \end{array}$$ (29)

From (27), (28) and (29), it is plainly clear that no known distribution can be analytically reduced to the conditional posterior distributions of parameters, and as a result, they cannot be sampled directly using the techniques that are generally accepted. Therefore, in order to mimic samples from (27), (28) and (29), the Metropolis-Hasting (MH) method using normal proposal distributions.

The MH technique is a very useful MCMC strategy since it can generate random samples from a posterior density distribution with an independent proposal distribution, as well as calculate Bayes estimates and generate HPD credible intervals. Furthermore, this technique provides an easy-to-apply chain form of the Bayesian estimate from a practical standpoint.

## 7 Simulations study and its numerical results

This section is devoted to exploring the performance of the introduced estimation methods for estimating the parameters of the proposed model using detailed simulation results. Several sample sizes, *n* = {50, 150}, and several values of the parameters, *α* = {0.25, 1.5}, *β* = {0.3, 1.3} and *c* = {0.6, 2}, are considered to generate *N* = 5000 random samples from the proposed distribution based on Eq (8). The average biases (*Bias*), and mean square errors (MSEs) are calculated. using the following equations

$$\begin{array}{c} Bias=\frac{1}{N}\sum_{i=1}^{N}\left|\hat{\Theta }-\Theta \right|,\;\;\;\;MSE=\frac{1}{N}\sum_{i=1}^{N}{\left(\hat{\Theta }-\Theta \right)}^{2} \end{array}$$

where **Θ** = *α*, *β*, *c*. The simulation results of the suggested model parameters using the nine estimation methodologies are shown in Tables 1–4.

**Table 1:** Bayesian ad non-Bayesian estimation for parameters of WPM distribution:A.

*α* = 1.5, *β* = 1.3			MLE					SELF			ELF d = -0.5			ELF d = 1.5		
c	n		Bias	MSE	L.CI	L.BP	L.BT	Bias	MSE	L.CCI	Bias	MSE	L.CCI	Bias	MSE	L.CCI
0.6	50	*α*	-0.1325	0.9573	3.8020	0.1266	0.1267	0.0162	0.0237	0.5757	0.0103	0.0234	0.5759	-0.0134	0.0230	0.5820
		*β*	0.1117	0.6226	3.0636	0.0942	0.0946	0.0257	0.0223	0.5537	0.0198	0.0219	0.5561	-0.0042	0.0210	0.5485
		c	0.0180	0.0480	0.8566	0.0266	0.0267	0.0255	0.0465	0.7895	-0.0041	0.0433	0.7890	-0.1625	0.0426	0.6907
		S(1)	0.1315	0.0805	0.9863	0.0311	0.0310	-0.0010	0.0021	0.1768	-0.0025	0.0021	0.1774	-0.0140	0.0026	0.1905
		S(1.5)	0.1485	0.0731	0.8864	0.0285	0.0286	-0.0006	0.0005	0.0803	0.0002	0.0005	0.0811	0.0024	0.0005	0.0829
		*S*_*F*_(*δ* = 0.25)	-0.0437	0.0048	0.2105	0.0064	0.0064	0.0020	0.0003	0.0610	0.0008	0.0003	0.0607	-0.0048	0.0003	0.0614
		*S*_*F*_(*δ* = 0.55)	-0.0562	0.0189	0.4917	0.0162	0.0163	0.0024	0.0009	0.1142	0.0003	0.0009	0.1143	-0.0109	0.0010	0.1179
		*S*_*F*_(*δ* = 0.9)	-0.0277	0.0447	0.8219	0.0244	0.0245	0.0003	0.0014	0.1455	-0.0020	0.0015	0.1466	-0.0158	0.0018	0.1537
	150	*α*	0.0363	0.5669	2.9495	0.0982	0.0989	0.0034	0.0109	0.4035	0.0019	0.0109	0.4012	-0.0039	0.0109	0.4016
		*β*	0.0200	0.1894	1.7052	0.0571	0.0562	0.0049	0.0082	0.3460	0.0036	0.0082	0.3453	-0.0020	0.0082	0.3496
		c	0.0304	0.0456	0.8286	0.0274	0.0271	-0.0104	0.0254	0.5995	-0.0173	0.0267	0.6207	-0.0479	0.0357	0.7175
		S(1)	0.03979	0.03984	0.76711	0.02401	0.02435	-0.00150	0.00098	0.12170	-0.00184	0.00099	0.12141	-0.00361	0.00107	0.12387
		S(1.5)	0.04436	0.01090	0.37057	0.01165	0.01164	0.00060	0.00022	0.05672	0.00081	0.00022	0.05689	0.00158	0.00023	0.05783
		*S*_*F*_(*δ* = 0.25)	-0.02000	0.00187	0.15023	0.00487	0.00483	-0.00021	0.00009	0.03771	-0.00048	0.00009	0.03779	-0.00167	0.00010	0.03806
		*S*_*F*_(*δ* = 0.55)	-0.02400	0.00926	0.36555	0.01188	0.01177	-0.00088	0.00035	0.07342	-0.00139	0.00035	0.07361	-0.00364	0.00038	0.07568
		*S*_*F*_(*δ* = 0.9)	-0.00963	0.02335	0.59817	0.01830	0.01832	-0.00189	0.00061	0.09624	-0.00246	0.00061	0.09668	-0.00504	0.00067	0.09981
2	50	*α*	-0.2009	0.9134	3.6646	0.1190	0.1155	0.0181	0.0288	0.6376	0.0117	0.0285	0.6369	-0.0140	0.0282	0.6345
		*β*	0.0992	0.6536	3.1467	0.0993	0.1001	0.0246	0.0229	0.5703	0.0195	0.0226	0.5696	-0.0013	0.0217	0.5642
		c	0.2127	1.1643	4.1489	0.1340	0.1343	0.0105	0.0872	1.1325	0.0005	0.0874	1.1316	-0.0402	0.0911	1.1451
		S(1)	0.15613	0.05591	0.69649	0.02265	0.02245	-0.00581	0.00192	0.16974	-0.00398	0.00191	0.17042	0.00340	0.00196	0.17061
		S(1.5)	0.26355	0.16286	1.19859	0.03819	0.03890	-0.00422	0.00109	0.12639	-0.00172	0.00111	0.12911	0.00901	0.00137	0.13775
		*S*_*F*_(*δ* = 0.25)	-0.06501	0.00804	0.24221	0.00759	0.00761	0.00244	0.00026	0.05993	0.00140	0.00025	0.05926	-0.00284	0.00025	0.05994
		*S*_*F*_(*δ* = 0.55)	-0.11464	0.03052	0.51705	0.01584	0.01580	0.00319	0.00099	0.11770	0.00159	0.00098	0.11714	-0.00515	0.00100	0.11659
		*S*_*F*_(*δ* = 0.9)	-0.11562	0.05515	0.80167	0.02591	0.02557	-0.00021	0.00151	0.15062	-0.00121	0.00151	0.15096	-0.00584	0.00157	0.15349
	150	*α*	0.0007	0.6245	3.0994	0.1006	0.1008	0.0057	0.0126	0.4274	0.0042	0.0125	0.4271	-0.0017	0.0126	0.4286
		*β*	0.0220	0.2335	1.8932	0.0591	0.0594	0.0060	0.0071	0.3155	0.0049	0.0070	0.3158	0.0001	0.0070	0.3167
		c	0.2048	1.1116	4.0562	0.1324	0.1322	-0.0074	0.0249	0.6126	-0.0092	0.0250	0.6138	-0.0162	0.0256	0.6161
		S(1)	0.07105	0.02651	0.57452	0.01867	0.01875	-0.00252	0.00082	0.10827	-0.00203	0.00082	0.10893	-0.00009	0.00083	0.11014
		S(1.5)	0.10817	0.04484	0.71400	0.02326	0.02318	-0.00088	0.00043	0.07965	-0.00026	0.00043	0.07979	0.00228	0.00045	0.08024
		*S*_*F*_(*δ* = 0.25)	-0.03092	0.00281	0.16906	0.00544	0.00544	0.00037	0.00008	0.03392	0.00013	0.00008	0.03386	-0.00084	0.00008	0.03410
		*S*_*F*_(*δ* = 0.55)	-0.05106	0.00975	0.33155	0.01033	0.01045	0.00012	0.00031	0.06822	-0.00025	0.00031	0.06826	-0.00174	0.00031	0.06797
		*S*_*F*_(*δ* = 0.9)	-0.04357	0.01708	0.48326	0.01574	0.01592	-0.00120	0.00053	0.08903	-0.00142	0.00053	0.08933	-0.00233	0.00054	0.08857

**Table 2:** Bayesian ad non-Bayesian estimation for parameters of WPM distribution:B.

*α* = 0.25, *β* = 1.3			MLE					SELF			ELF d = -0.5			ELF d = 1.5		
c	n		Bias	MSE	L.CI	L.BP	L.BT	Bias	MSE	L.CCI	Bias	MSE	L.CCI	Bias	MSE	L.CCI
0.6	50	*α*	-0.0614	0.0273	0.6017	0.0184	0.0183	0.0286	0.0075	0.2973	0.0210	0.0068	0.2894	-0.0079	0.0054	0.2657
		*β*	0.1675	0.6440	3.0781	0.0965	0.0973	-0.0019	0.0212	0.5390	-0.0074	0.0212	0.5382	-0.0297	0.0218	0.5398
		c	0.0456	0.0607	0.9492	0.0295	0.0291	0.0297	0.0567	0.8963	0.0022	0.0528	0.8697	-0.1396	0.5108	0.8981
		S(1)	0.01883	0.00177	0.14744	0.00458	0.00463	-0.00727	0.00044	0.07285	-0.00606	0.00041	0.07252	-0.00326	0.00041	0.07391
		S(1.5)	0.11022	0.02658	0.47117	0.01532	0.01538	-0.01980	0.00254	0.17563	-0.01248	0.00224	0.17154	0.01103	0.00245	0.18374
		*S*_*F*_(*δ* = 0.25)	-0.01468	0.00050	0.06636	0.00203	0.00203	0.00280	0.00006	0.02743	0.00180	0.00005	0.02648	-0.00132	0.00005	0.02699
		*S*_*F*_(*δ* = 0.55)	-0.03961	0.00330	0.16315	0.00533	0.00535	0.00659	0.00031	0.06204	0.00390	0.00027	0.06047	-0.00483	0.00029	0.06212
		*S*_*F*_(*δ* = 0.9)	-0.07857	0.01185	0.29554	0.00961	0.00963	0.01124	0.00082	0.10224	0.00595	0.00070	0.09889	-0.01179	0.00088	0.10538
	150	*α*	-0.0400	0.0194	0.5228	0.0160	0.0159	0.0052	0.0021	0.1759	0.0035	0.0020	0.1757	-0.0033	0.0019	0.1726
		*β*	0.0612	0.2024	1.7480	0.0553	0.0555	0.0040	0.0080	0.3447	0.0027	0.0080	0.3449	-0.0022	0.0080	0.3475
		c	0.0312	0.0520	0.8861	0.0292	0.0282	-0.0078	0.0249	0.6187	-0.0141	0.0259	0.6330	-0.0410	0.0322	0.6753
		S(1)	0.01176	0.00129	0.13334	0.00417	0.00406	-0.00217	0.00015	0.04593	-0.00190	0.00015	0.04599	-0.00092	0.00015	0.04615
		S(1.5)	0.07171	0.01666	0.42097	0.01355	0.01354	-0.00496	0.00084	0.10919	-0.00328	0.00083	0.10989	0.00315	0.00087	0.11274
		*S*_*F*_(*δ* = 0.25)	-0.00968	0.00033	0.06041	0.00189	0.00183	0.00068	0.00002	0.01709	0.00046	0.00002	0.01701	-0.00041	0.00002	0.01694
		*S*_*F*_(*δ* = 0.55)	-0.02616	0.00210	0.14743	0.00468	0.00464	0.00154	0.00010	0.03821	0.00092	0.00010	0.03803	-0.00144	0.00011	0.03898
		*S*_*F*_(*δ* = 0.9)	-0.05167	0.00717	0.26313	0.00840	0.00836	0.00252	0.00026	0.06082	0.00130	0.00026	0.06084	-0.00339	0.00028	0.06258
2	50	*α*	-0.0635	0.0280	0.6075	0.0189	0.0190	0.0404	0.0118	0.3739	0.0312	0.0105	0.3600	-0.0039	0.0076	0.3245
		*β*	0.1555	0.6910	3.2027	0.1063	0.1045	-0.0056	0.0255	0.6331	-0.0110	0.0255	0.6338	-0.0328	0.0261	0.6328
		c	0.1899	1.0743	3.9961	0.1268	0.1269	0.0096	0.0875	1.1290	0.0012	0.0874	1.1243	-0.0327	0.0891	1.1325
		S(1)	0.00843	0.00015	0.03537	0.00115	0.00114	-0.00461	0.00011	0.03293	-0.00339	0.00008	0.02987	0.00077	0.00005	0.02415
		S(1.5)	0.07063	0.00784	0.20945	0.00661	0.00654	-0.02081	0.00170	0.13484	-0.01241	0.00128	0.12537	0.01678	0.00118	0.11355
		*S*_*F*_(*δ* = 0.25)	-0.00787	0.00010	0.02515	0.00081	0.00080	0.00283	0.00004	0.02000	0.00182	0.00003	0.01810	-0.00163	0.00002	0.01549
		*S*_*F*_(*δ* = 0.55)	-0.02333	0.00087	0.07041	0.00216	0.00217	0.00738	0.00023	0.05043	0.00450	0.00017	0.04624	-0.00540	0.00014	0.04064
		*S*_*F*_(*δ* = 0.9)	-0.05179	0.00412	0.14874	0.00464	0.00461	0.01408	0.00077	0.09084	0.00800	0.00057	0.08575	-0.01308	0.00059	0.07894
	150	*α*	-0.0443	0.0208	0.5383	0.0163	0.0163	0.0104	0.0030	0.2037	0.0082	0.0029	0.2036	-0.0003	0.0026	0.2002
		*β*	0.0578	0.2492	1.9448	0.0620	0.0606	-0.0014	0.0086	0.3516	-0.0026	0.0086	0.3511	-0.0076	0.0086	0.3530
		c	0.1060	1.0569	4.0106	0.1223	0.1194	0.0032	0.0240	0.5814	0.0016	0.0240	0.5828	-0.0047	0.0243	0.5867
		S(1)	0.00601	0.00009	0.02843	0.00095	0.00095	-0.00113	0.00002	0.01652	-0.00086	0.00002	0.01626	0.00015	0.00002	0.01542
		S(1.5)	0.04998	0.00495	0.19409	0.00612	0.00610	-0.00469	0.00039	0.07358	-0.00274	0.00036	0.07215	0.00478	0.00036	0.07167
		*S*_*F*_(*δ* = 0.25)	-0.00563	0.00006	0.02253	0.00070	0.00070	0.00066	0.00001	0.01050	0.00043	0.00001	0.01028	-0.00045	0.00001	0.01017
		*S*_*F*_(*δ* = 0.55)	-0.01662	0.00055	0.06474	0.00208	0.00206	0.00168	0.00005	0.02726	0.00101	0.00005	0.02658	-0.00154	0.00005	0.02625
		*S*_*F*_(*δ* = 0.9)	-0.03668	0.00262	0.14000	0.00452	0.00457	0.00312	0.00018	0.04952	0.00169	0.00017	0.04935	-0.00378	0.00017	0.04927

**Table 3:** Bayesian ad non-Bayesian estimation for parameters of WPM distribution:C.

*α* = 1.5, *β* = 0.3			MLE					SELF			ELF d = -0.5			ELF d = 1.5		
c	n		Bias	MSE	L.CI	L.BP	L.BT	Bias	MSE	L.CCI	Bias	MSE	L.CCI	Bias	MSE	L.CCI
0.6	50	*α*	-0.2148	0.9269	3.6808	0.1133	0.1130	-0.0145	0.0376	0.7236	-0.0218	0.0378	0.7217	-0.0516	0.0397	0.7223
		*β*	0.0232	0.0329	0.7055	0.0219	0.0216	0.0075	0.0010	0.1146	0.0062	0.0010	0.1142	0.0009	0.0009	0.1115
		c	0.0520	0.1103	1.2864	0.0411	0.0415	-0.0097	0.0267	0.6311	-0.0247	0.0275	0.6391	-0.0990	0.0434	0.7312
		S(2)	0.23272	0.10312	0.86783	0.02741	0.02725	-0.00745	0.00282	0.20940	-0.00899	0.00287	0.20780	-0.02224	0.00417	0.23302
		S(10)	0.24366	0.17708	1.34554	0.04307	0.04318	-0.00059	0.00032	0.06534	0.00022	0.00033	0.06573	0.00269	0.00037	0.07103
		*S*_*F*_(*δ* = 0.25)	-0.05652	0.01340	0.39629	0.01218	0.01235	-0.00018	0.00055	0.09190	-0.00223	0.00056	0.09306	-0.01310	0.00082	0.10291
		*S*_*F*_(*δ* = 0.55)	-0.04476	0.03346	0.69556	0.02158	0.02150	-0.00348	0.00127	0.14128	-0.00609	0.00130	0.14011	-0.02132	0.00208	0.16122
		*S*_*F*_(*δ* = 0.9)	-0.01740	0.05347	0.90433	0.02831	0.02799	-0.00635	0.00185	0.16838	-0.00879	0.00190	0.16944	-0.02461	0.00302	0.19327
	150	*α*	-0.0390	0.6496	3.1572	0.1046	0.1043	-0.0094	0.0177	0.5138	-0.0111	0.0178	0.5159	-0.0179	0.0183	0.5253
		*β*	0.0053	0.0131	0.4481	0.0141	0.0140	0.0033	0.0004	0.0789	0.0029	0.0004	0.0790	0.0014	0.0004	0.0785
		c	0.0301	0.1034	1.2558	0.0422	0.0424	-0.0041	0.0110	0.3982	-0.0075	0.0111	0.4000	-0.0215	0.0123	0.4126
		S(2)	0.11989	0.04708	0.70928	0.02340	0.02379	-0.00178	0.00117	0.12896	-0.00197	0.00118	0.12936	-0.00291	0.00125	0.13368
		S(10)	0.11197	0.05931	0.84818	0.02586	0.02589	-0.00021	0.00012	0.04293	0.00006	0.00013	0.04305	0.00117	0.00013	0.04384
		*S*_*F*_(*δ* = 0.25)	-0.02575	0.00523	0.26500	0.00798	0.00797	0.00036	0.00025	0.06178	-0.00015	0.00026	0.06176	-0.00225	0.00027	0.06176
		*S*_*F*_(*δ* = 0.55)	-0.01534	0.01285	0.44043	0.01419	0.01417	-0.00047	0.00059	0.09382	-0.00106	0.00060	0.09397	-0.00358	0.00064	0.09584
		*S*_*F*_(*δ* = 0.9)	0.00332	0.02041	0.56013	0.01809	0.01821	-0.00139	0.00083	0.10827	-0.00188	0.00084	0.10844	-0.00406	0.00090	0.11195
2	50	*α*	-0.4102	0.9557	3.4802	0.1039	0.1045	-0.0092	0.0408	0.7662	-0.0156	0.0410	0.7697	-0.0413	0.0430	0.7771
		*β*	0.0376	0.0414	0.7846	0.0244	0.0244	0.0031	0.0005	0.0915	0.0026	0.0005	0.0914	0.0005	0.0005	0.0900
		c	0.3306	2.3587	5.8822	0.1774	0.1807	-0.0156	0.0615	0.9624	-0.0214	0.0618	0.9641	-0.0445	0.0641	0.9645
		S(2)	0.04931	0.00513	0.20366	0.00638	0.00636	-0.00537	0.00067	0.09699	-0.00422	0.00065	0.09710	0.00024	0.00062	0.09555
		S(10)	0.36604	0.25682	1.37459	0.04155	0.04160	-0.00691	0.00242	0.18872	-0.00244	0.00238	0.19140	0.01620	0.00285	0.19747
		*S*_*F*_(*δ* = 0.25)	-0.10692	0.02050	0.37352	0.01175	0.01172	0.00338	0.00057	0.09197	0.00129	0.00055	0.09140	-0.00708	0.00061	0.09037
		*S*_*F*_(*δ* = 0.55)	-0.21901	0.08807	0.78539	0.02583	0.02577	0.00412	0.00141	0.14646	0.00068	0.00138	0.14651	-0.01342	0.00161	0.14554
		*S*_*F*_(*δ* = 0.9)	-0.30153	0.17258	1.12077	0.03627	0.03629	0.00229	0.00171	0.16092	-0.00119	0.00170	0.16031	-0.01592	0.00206	0.16161
	150	*α*	-0.2385	0.6946	3.1321	0.0996	0.0985	-0.0095	0.0180	0.5203	-0.0111	0.0181	0.5191	-0.0175	0.0185	0.5187
		*β*	0.0104	0.0172	0.5130	0.0159	0.0160	0.0017	0.0002	0.0580	0.0016	0.0002	0.0579	0.0010	0.0002	0.0580
		c	0.0445	1.2818	4.4368	0.1330	0.1333	-0.0080	0.0165	0.4952	-0.0091	0.0166	0.4941	-0.0137	0.0168	0.4983
		S(2)	0.03056	0.00318	0.18585	0.00615	0.00610	-0.00133	0.00019	0.05321	-0.00099	0.00019	0.05330	0.00033	0.00019	0.05338
		S(10)	0.23925	0.17037	1.31914	0.04123	0.04135	-0.00192	0.00082	0.10897	-0.00066	0.00081	0.10963	0.00443	0.00085	0.10990
		*S*_*F*_(*δ* = 0.25)	-0.06890	0.01296	0.35540	0.01123	0.01126	0.00085	0.00018	0.05038	0.00026	0.00018	0.05045	-0.00208	0.00018	0.05114
		*S*_*F*_(*δ* = 0.55)	-0.14184	0.05662	0.74932	0.02400	0.02466	0.00117	0.00048	0.08378	0.00022	0.00047	0.08423	-0.00365	0.00049	0.08466
		*S*_*F*_(*δ* = 0.9)	-0.19768	0.11423	1.07514	0.03302	0.03364	0.00079	0.00062	0.09341	-0.00018	0.00061	0.09298	-0.00412	0.00063	0.09544

**Table 4:** Bayesian ad Non-Bayesian estimation for parameters of WPM distribution:D.

*α* = 0.25, *β* = 0.3			MLE					SELF			ELF d = -0.5			ELF d = 1.5		
c	n		Bias	MSE	L.CI	L.BP	L.BT	Bias	MSE	L.CCI	Bias	MSE	L.CCI	Bias	MSE	L.CCI
0.6	50	*α*	-0.0701	0.0291	0.6098	0.0201	0.0200	0.0290	0.0167	0.4479	0.0159	0.0152	0.4353	-0.0385	0.0128	0.3579
		*β*	0.0439	0.0357	0.7205	0.0222	0.0226	0.0090	0.0017	0.1551	0.0073	0.0016	0.1543	0.0005	0.0015	0.1475
		c	0.0549	0.1053	1.2541	0.0416	0.0415	-0.0086	0.0426	0.7584	-0.0246	0.0446	0.7705	-0.1015	0.0673	0.8542
		S(2)	0.01265	0.00029	0.04444	0.00139	0.00138	-0.00682	0.00022	0.04705	-0.00509	0.00018	0.04587	-0.00020	0.00020	0.04664
		S(50)	0.32303	0.17017	1.00620	0.03187	0.03193	-0.06376	0.00752	0.23452	-0.03079	0.00401	0.22014	0.11459	0.02153	0.35526
		*S*_*F*_(*δ* = 0.25)	-0.11810	0.02146	0.34001	0.01066	0.01060	0.03379	0.00273	0.15564	0.01660	0.00168	0.14779	-0.04365	0.00404	0.17644
		*S*_*F*_(*δ* = 0.55)	-0.22417	0.07883	0.66304	0.02045	0.02062	0.05275	0.00556	0.21130	0.02439	0.00296	0.19342	-0.08601	0.01228	0.26886
		*S*_*F*_(*δ* = 0.9)	-0.29696	0.14279	0.91648	0.03060	0.02988	0.05750	0.00635	0.21915	0.02605	0.00328	0.20274	-0.11107	0.01893	0.31606
	150	*α*	-0.0484	0.0207	0.5318	0.0170	0.0173	0.0052	0.0050	0.2645	0.0014	0.0049	0.2632	-0.0140	0.0049	0.2629
		*β*	0.0138	0.0130	0.4432	0.0139	0.0138	0.0045	0.0007	0.0990	0.0040	0.0007	0.0983	0.0019	0.0006	0.0959
		c	0.0208	0.1015	1.2468	0.0396	0.0398	-0.0127	0.0131	0.4231	-0.0162	0.0135	0.4288	-0.0306	0.0156	0.4569
		S(2)	0.00853	0.00019	0.04332	0.00140	0.00140	-0.00210	0.00005	0.02469	-0.00152	0.00005	0.02493	0.00070	0.00004	0.02503
		S(50)	0.22359	0.11177	0.97483	0.03174	0.03149	-0.01947	0.00153	0.12620	-0.00863	0.00114	0.12543	0.03700	0.00319	0.16321
		*S*_*F*_(*δ* = 0.25)	-0.08276	0.01429	0.33827	0.01080	0.01066	0.00977	0.00060	0.08836	0.00444	0.00050	0.08703	-0.01632	0.00089	0.10072
		*S*_*F*_(*δ* = 0.55)	-0.15616	0.05182	0.64958	0.02018	0.02018	0.01548	0.00112	0.11477	0.00653	0.00086	0.11207	-0.02957	0.00215	0.14080
		*S*_*F*_(*δ* = 0.9)	-0.20587	0.09360	0.88763	0.02811	0.02817	0.01739	0.00129	0.11719	0.00716	0.00096	0.11396	-0.03572	0.00280	0.14801
2	50	*α*	-0.0753	0.0281	0.5879	0.0181	0.0184	0.0255	0.0154	0.4354	0.0118	0.0144	0.4246	-0.0468	0.0146	0.3932
		*β*	0.0308	0.0249	0.6074	0.0194	0.0195	0.0100	0.0015	0.1461	0.0085	0.0014	0.1437	0.0032	0.0013	0.1367
		c	0.4762	2.8890	6.3991	0.2088	0.2074	-0.0149	0.0504	0.8568	-0.0187	0.0507	0.8568	-0.0342	0.0524	0.8648
		S(20)	0.00933	0.00036	0.06531	0.00202	0.00202	-0.01355	0.00041	0.05551	-0.00559	0.00016	0.04235	0.01316	0.00024	0.02570
		S(150)	0.27211	0.15999	1.14978	0.03689	0.03696	-0.10071	0.01544	0.28527	-0.04346	0.00541	0.23669	0.23508	0.07166	0.48916
		*S*_*F*_(*δ* = 0.25)	-0.06427	0.00837	0.25547	0.00830	0.00839	0.04654	0.00367	0.14560	0.01789	0.00112	0.10867	-0.06834	0.00577	0.11841
		*S*_*F*_(*δ* = 0.55)	-0.15744	0.05032	0.62663	0.01880	0.01935	0.08290	0.01089	0.24663	0.03344	0.00341	0.18946	-0.15323	0.02929	0.28325
		*S*_*F*_(*δ* = 0.9)	-0.24366	0.12528	1.00688	0.03202	0.03226	0.08970	0.01253	0.26308	0.03839	0.00447	0.21455	-0.21281	0.05825	0.43457
	150	*α*	-0.0623	0.0219	0.5258	0.0160	0.0158	0.0110	0.0063	0.2858	0.0070	0.0062	0.2861	-0.0096	0.0061	0.2853
		*β*	0.0142	0.0129	0.4414	0.0138	0.0138	0.0030	0.0006	0.0941	0.0026	0.0006	0.0939	0.0011	0.0006	0.0934
		c	0.1870	1.6674	5.0110	0.1581	0.1555	-0.0032	0.0124	0.4311	-0.0040	0.0124	0.4315	-0.0072	0.0125	0.4345
		S(20)	0.00634	0.00025	0.05657	0.00179	0.00180	-0.00338	0.00006	0.02600	-0.00155	0.00005	0.02479	0.00469	0.00006	0.02444
		S(150)	0.21429	0.12553	1.10657	0.03458	0.03457	-0.02865	0.00213	0.14234	-0.01103	0.00114	0.12597	0.06568	0.00768	0.21947
		*S*_*F*_(*δ* = 0.25)	-0.04850	0.00627	0.24559	0.00782	0.00781	0.01168	0.00043	0.06791	0.00429	0.00027	0.05983	-0.02311	0.00102	0.08487
		*S*_*F*_(*δ* = 0.55)	-0.12192	0.03862	0.60445	0.01942	0.01933	0.02199	0.00131	0.11475	0.00804	0.00072	0.10131	-0.04779	0.00406	0.16192
		*S*_*F*_(*δ* = 0.9)	-0.19207	0.09798	0.96932	0.03035	0.02997	0.02550	0.00175	0.12775	0.00952	0.00093	0.11167	-0.06016	0.00636	0.19831

It is concluded from the Tables 1–4 that all of the suggested distribution’s parameter estimates are extremely reliable and very close to their true values, with minimal biases, and MSEs in all circumstances. For all scenarios analysed, the proposed estimators are consistent, with the bias and MSE decreasing as *n* grows. The MLE and Bayesian estimate approaches based on symmetric and asymmetric loss functions perform admirably in estimating the proposed model parameters.

## 8 Modeling real data with analysis

In this section, we focus on three real data analyses which are relevant to Covid-19. The first data is taken from [29] and represents the United Kingdom transformed Covid-19 data and spans 82 days, from May 1, 2021 to July 16, 2021. For the first dataset, the transformation was performed by [29] according to the following procedure:

$$\begin{array}{c} x_{i}=\frac{ND_{i}}{CC_{i}-CD_{i-1}}\times 1000, \end{array}$$

where ND is daily new deaths, CC is daily cumulative cases, CD is daily cumulative deaths and *x*_*i*_ is the transformed data. The first data is given as folllows: 0.0023, 0.0023, 0.0023, 0.0046, 0.0065, 0.0067, 0.0069, 0.0069, 0.0091, 0.0093, 0.0093, 0.0093, 0.0111, 0.0115, 0.0116, 0.0116, 0.0119, 0.0133, 0.0136, 0.0138, 0.0138, 0.0159, 0.0161, 0.0162, 0.0162, 0.0162, 0.0163, 0.0180, 0.0187, 0.0202, 0.0207, 0.0208, 0.0225, 0.0230, 0.0230, 0.0239, 0.0245, 0.0251, 0.0255, 0.0255, 0.0271, 0.0275, 0.0295, 0.0297, 0.0300, 0.0302, 0.0312, 0.0314, 0.0326, 0.0346, 0.0349, 0.0350, 0.0355, 0.0379, 0.0384, 0.0394, 0.0394, 0.0412, 0.0419, 0.0425, 0.0461, 0.0464, 0.0468, 0.0471, 0.0495, 0.0501, 0.0521, 0.0571, 0.0588, 0.0597, 0.0628, 0.0679, 0.0685, 0.0715, 0.0766, 0.0780, 0.0942, 0.0960, 0.0988, 0.1223, 0.1343, and 0.1781. The second data representing the drought mortality rate from COVID-19 owned by Canada for 36 days, April 10, 2020 to May 15, 2020, see link https://covid19.who.int/ or [30]. The second data is given as follows: 3.1091, 3.3825, 3.1444, 3.2135, 2.4946, 3.5146, 4.9274, 3.3769, 6.8686, 3.0914, 4.9378, 3.1091, 3.2823, 3.8594, 4.0480, 4.1685, 3.6426, 3.2110, 2.8636, 3.2218, 2.9078, 3.6346, 2.7957, 4.2781, 4.2202, 1.5157, 2.6029, 3.3592, 2.8349, 3.1348, 2.5261, 1.5806, 2.7704, 2.1901, 2.4141 and 1.9048.

The third data represents the COVID-19 data of Saudi Arabia for a period of 283 days from April 20, 2020, to January 17, 2021, which represents the number of deaths per day and is derived from [8]. Third data is given as follows: 0.0831716, 0.125439, 0.15873, 0.123077, 0.15083, 0.103645, 0.063482, 0.0566653, 0.0610724, 0.0539374, 0.0577645, 0.0520472, 0.0394851, 0.0506659, 0.0400507, 0.0556517, 0.0172553, 0.0267781, 0.0401345, 0.0235294, 0.0221278, 0.0292459, 0.0276789, 0.0298118, 0.0245856, 0.0299401, 0.0283572, 0.0298312, 0.0283986, 0.0270951, 0.0180371, 0.0220761, 0.0210921, 0.0201948, 0.0214611, 0.0184075, 0.0193334, 0.0183655, 0.014027, 0.0151174, 0.016073, 0.0185363, 0.019297, 0.0214909, 0.0152395, 0.012096, 0.0157218, 0.0179189, 0.0200602, 0.0209038, 0.0265297, 0.0271287, 0.0253927, 0.0271229, 0.0331005, 0.0345654, 0.0325846, 0.0346137, 0.0355598, 0.0325138, 0.0343149, 0.0322855, 0.0329855, 0.0302305, 0.0318588, 0.0315933, 0.0297537, 0.0303021, 0.027821, 0.0331263, 0.0301704, 0.0300557, 0.02366, 0.025041, 0.0239503, 0.0247106, 0.0242242, 0.0265667, 0.0209004, 0.0221016, 0.0259621, 0.0264237, 0.0254445, 0.0275637, 0.0249939, 0.0274339, 0.0279315, 0.0245515, 0.0227759, 0.0192548, 0.0185296, 0.0227287, 0.0131967, 0.0182547, 0.00858782, 0.0169808, 0.0176329, 0.0186789, 0.0151963, 0.0162596, 0.0156959, 0.0147491, 0.0134227, 0.0172145, 0.0131889, 0.0142233, 0.0118185, 0.0113534, 0.0101427, 0.010818, 0.0100074, 0.00957985, 0.00878918, 0.00764715, 0.0108716, 0.011545, 0.012567, 0.0128645, 0.0124464, 0.01344, 0.0130204, 0.012957, 0.0111584, 0.0117945, 0.0124222, 0.0116738, 0.0119618, 0.010545, 0.0132126, 0.00944332, 0.0114137, 0.0120314, 0.0139782, 0.0106087, 0.0128802, 0.00987297, 0.0137703, 0.0101282, 0.0107452, 0.0097371, 0.00905741, 0.00870692, 0.00964689, 0.00865657, 0.0102311, 0.00861087, 0.00827069, 0.0104709, 0.0107623, 0.0101061, 0.0081922, 0.00943011, 0.00878087, 0.00751044, 0.00749489, 0.00841549, 0.00871159, 0.011491, 0.0102286, 0.0095912, 0.00926569, 0.00955843, 0.0086196, 0.00830013, 0.00828828, 0.00919439, 0.0082615, 0.00916624, 0.00793336, 0.00914174, 0.00852261, 0.00881553, 0.00819486, 0.00879145, 0.00787094, 0.00876705, 0.00815281, 0.00754064, 0.00692999, 0.0075223, 0.0072118, 0.00750334, 0.00719496, 0.00658785, 0.00747945, 0.00747223, 0.00567129, 0.00625926, 0.00565554, 0.00505399, 0.00623682, 0.00593407, 0.00474217, 0.00473699, 0.00532299, 0.0044308, 0.00413093, 0.00501049, 0.00441704, 0.00500094, 0.00470149, 0.00557646, 0.0043971, 0.00585622, 0.0055572, 0.00525925, 0.00496181, 0.00553818, 0.00436707, 0.00523385, 0.00493708, 0.00551167, 0.00434698, 0.00550017, 0.00491477, 0.00404305, 0.00432813, 0.00576376, 0.00460664, 0.00460281, 0.00546136, 0.00459451, 0.00516476, 0.00544698, 0.00458342, 0.00458073, 0.00543638, 0.00457523, 0.00428639, 0.00399708, 0.00427882, 0.00484536, 0.00370313, 0.00398567, 0.00341417, 0.00312743, 0.00340943, 0.00312338, 0.00283764, 0.00397066, 0.00311828, 0.00339985, 0.0033981, 0.00367975, 0.00282955, 0.00311111, 0.00367516, 0.00339125, 0.00310765, 0.00282409, 0.00310501, 0.00310352, 0.00282009, 0.0031008, 0.00281771, 0.00253481, 0.00225207, 0.00253238, 0.00309356, 0.00252991, 0.00224784, 0.00252778, 0.00308855, 0.00224535, 0.00280586, 0.00252435, 0.00196268, 0.00252278, 0.00196176, 0.00280183, 0.00252099, 0.00196017, 0.00167967, 0.00111949, 0.00111916, 0.00139851, 0.00111839, 0.00139742, 0.00111741, 0.00167534, 0.00083728, 0.00139493, 0.00139427.

For the comparison, it is considered Maxwell (M), PM, inverse Maxwell (IM) [31], weighted Maxwell-Boltzmann (WMB) [32], length-biased Maxwell (LBM) [33], generalized Maxwell failure (GMF) [12] and Weibull (W) distributions. The all PDFs of these distributions are presented in Table 5. Table 5 gives information about the parameters of the distributions reported in Tables 6 and 7. The MLEs of parameters, standard (SE) of MLEs, log-likelihood value (*ℓ*), Akaike’s information criteria (AIC), Bayesian information criterion (BIC), consistent AIC (CAIC), Hannan-Quinn information criterion (HQIC), Kolmogorov-Smirnov statistics (KS), Anderson-Darling statistics (AD), Cramér von Mises statistic (CvM) and related p-values (KS p-value, AD p-value and CvM p-value) are obtained and given in Tables 6–8.

**Table 5:** List of the distributions to modelling three real data.

$f_{WPM}(p_{1},p_{2},p_{3})=\frac{2p_{2}p_{1}^{\frac{1}{2}(\frac{p_{3}}{p_{2}}+3)}x^{3p_{2}+p_{3}-1}\text{exp}(-p_{1}x^{2p_{2}})}{\Gamma (\frac{1}{2}(\frac{p_{3}}{p_{2}}+3))}$	,	*p*_1_, *p*_2_, *p*_3_ > 0
$f_{M}(p_{1})=\frac{4}{\sqrt{\Pi }}p_{1}^{\frac{3}{2}}x^{2}\text{exp}(-p_{1}x^{2})$	,	*p*_1_ > 0
$f_{PM}(p_{1},p_{2})=\frac{4}{\sqrt{\Pi }}p_{1}^{\frac{3}{2}}p_{2}x^{3p_{2}-1}\text{exp}(-p_{1}x^{2p_{2}})$	,	*p*_1_, *p*_2_ > 0
$f_{IM}(p_{1})=\sqrt{\frac{2}{\Pi }}\text{exp}(-\frac{1}{2x^{2}p_{1}^{2}})p_{1}^{-3}x^{-4}$	,	*p*_1_ > 0
$f_{WMB}(p_{1},p_{2})=\frac{p_{1}^{\frac{p_{2}+3}{2}}x^{p_{2}+2}\text{exp}(-\frac{p_{1}x^{2}}{2})}{2^{\frac{p_{2+1}}{2}}\Gamma (\frac{p_{2}+3}{2})}$	,	*p*_1_, *p*_2_ > 0
$f_{LBM}(p_{1})=\frac{x^{3}\text{exp}(-\frac{x^{2}}{2p_{1}^{2}})}{2p_{1}^{4}}$	,	*p*_1_ > 0
$f_{GMF}(p_{1},p_{2})=\frac{2x^{2p_{1}-1}\text{exp}(-\frac{x^{2}}{p_{2}})}{p_{2}^{p_{1}}\Gamma (p_{1})}$	,	*p*_1_, *p*_2_ > 0
$f_{W}(p_{1},p_{2})=\frac{p_{1}}{p_{2}}{\left(\frac{x}{p_{2}}\right)}^{p_{1}-1}\text{exp}(-{\left(\frac{x}{p_{2}}\right)}^{p_{1}})$	,	*p*_1_, *p*_2_ > 0

**Table 6:** Data analysis results for first real data.

	WPM	M	PM	IM	WHB	LBM	GMF	W
*ℓ*	196.4810	139.8092	195.6106	60.0548	139.8092	100.7565	190.5852	194.5410
AIC	−386.9620	−277.6185	−387.2212	−118.1096	−275.6185	−199.5130	−377.1703	−385.0819
BIC	−379.7418	−275.2118	−382.4077	−115.7029	−270.8051	−197.1063	−372.3569	−380.2685
CAIC	−386.6543	−277.5685	−387.0693	−118.0596	−275.4666	−199.4630	−377.0184	−384.9300
HQIC	−384.0632	−276.6522	−385.2887	−117.1433	−273.6860	−198.5467	−375.2378	−383.1494
KS	0.0385	0.3205	0.0467	0.5233	0.3205	0.3725	0.1015	0.0572
AD	0.1255	27.1815	0.2543	78.5654	27.1815	43.5924	1.3417	0.4293
CvM	0.0135	3.4200	0.0334	9.4755	3.4200	4.5314	0.2426	0.0591
KS p-value	0.9997	0.0000	0.9939	0.0000	0.0000	0.0000	0.3673	0.9512
AD p-value	0.9997	0.0000	0.9680	0.0000	0.0000	0.0000	0.2192	0.8188
CVM p-value	0.9999	0.0000	0.9645	0.0000	0.0000	0.0000	0.1982	0.8207
*p*_1_	31.3388	668.7853	42.3337	59.8615	1337.5706	0.0237	0.5011	1.2518
*p*_2_	0.2731		0.5013		0.0000		0.0045	0.0386
*p*_3_	1.7379							
SE *p*_1_	5.1689	60.3029	11.1025	2.6988	225.1756	0.0009	0.0597	0.1028
SE *p*_2_	0.1659		0.0408		0.4265		0.0007	0.0036
SE *p*_3_	1.8862							

**Table 7:** Data analysis results for second real data.

	WPM	M	PM	IM	WHB	LBM	GMF	W
*ℓ*	−48.1547	−53.3111	−50.3062	−53.9296	−49.1169	−50.7361	−49.1169	−51.4743
AIC	102.3094	108.6223	104.6123	109.8592	102.2338	103.4721	102.2338	106.9485
BIC	107.0600	110.2058	107.7793	111.4428	105.4008	105.0556	105.4008	110.1156
CAIC	103.0594	108.7399	104.9759	109.9769	102.5974	103.5897	102.5974	107.3122
HQIC	103.9675	109.1750	105.7177	110.4119	103.3392	104.0248	103.3392	108.0539
KS	0.1071	0.2042	0.1437	0.1993	0.1359	0.1537	0.1359	0.1500
AD	0.5429	2.2009	0.9102	2.2135	0.6487	1.3162	0.6487	1.1423
CvM	0.0918	0.4049	0.1601	0.3964	0.1142	0.2364	0.1142	0.1979
KS p-value	0.8034	0.0994	0.4469	0.1146	0.5188	0.3629	0.5188	0.3929
AD p-value	0.7023	0.0718	0.4073	0.0707	0.6020	0.2270	0.6020	0.2902
CVM p-value	0.6293	0.0696	0.3615	0.0733	0.5218	0.2070	0.5218	0.2723
*p*_1_	37.5957	0.1278	0.0472	0.2012	0.5232	1.7131	3.0709	3.3136
*p*_2_	0.2166		1.3679		3.1417		3.8224	3.6370
*p*_3_	26.3258							
SE *p*_1_	65.5064	0.0174	0.0215	0.0137	0.1274	0.1009	0.6881	0.3789
SE *p*_2_	0.1508		0.1564		1.3762		0.9305	0.1941
SE *p*_3_	19.6732							

**Table 8:** Data analysis results for second third data.

	WPM	M	PM	IM	WHB	LBM	GMF	W
*ℓ*	909.9188	470.2825	893.3490	565.6476	470.2825	243.7173	843.6761	885.7773
AIC	−1813.8376	−938.5649	−1782.6981	−1129.2952	−936.5649	−485.4346	−1683.3523	−1767.5547
BIC	−1802.9012	−934.9195	−1775.4072	−1125.6497	−929.2740	−481.7892	−1676.0614	−1760.2638
CAIC	−1813.7515	−938.5507	−1782.6552	−1129.2809	−936.5221	−485.4204	−1683.3094	−1767.5118
HQIC	−1809.4525	−937.1032	−1779.7747	−1127.8335	−933.6415	−483.9729	−1680.4289	−1764.6313
KS	0.0453	0.4829	0.0648	0.4528	0.4829	0.5333	0.1469	0.0801
AD	0.6695	-	2.4784	187.8935	-	-	13.5133	3.5806
CvM	0.0976	-	0.3148	20.8777	25.4993	-	2.3170	0.4565
KS p-value	0.6077	0.0000	0.1856	0.0000	0.0000	0.0000	0.0000	0.0529
AD p-value	0.5847	-	0.0509	0.0000	0.0000	-	0.0000	0.0140
CVM p-value	0.5973	-	0.1207	0.0000	0.0000	-	0.0000	0.0514
*p*_1_	181.6447	2244.7609	49.3818	135.5863	4489.5218	0.0129	0.3246	1.0157
*p*_2_	0.0470		0.4147		0.0000		0.0022	0.0162
*p*_3_	10.9789							
SE *p*_1_	32.4799	108.9430	6.9767	3.2904	405.4450	0.0003	0.0186	0.0427
SE *p*_2_	0.0049		0.0174		0.2290		0.0001	0.0010
SE *p*_3_	1.4407							

According to results in Tables 6–8, it is seen that the WPM has minimum KS, AD and CvM and has the highest *ℓ* values. Hence, it can be concluded that the WPM can be a good alternative to modeling Covid-19 data sets.

**Fig 5:**
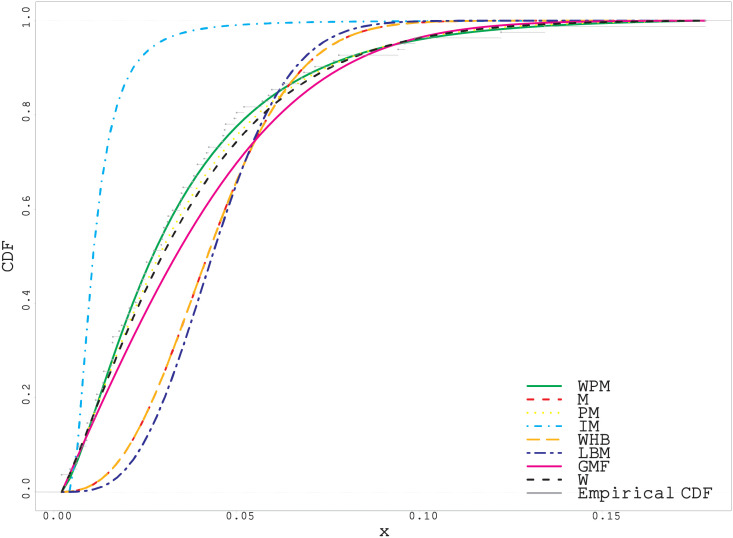
Fitted and empirical CDF plots for first real data.

**Fig 6:**
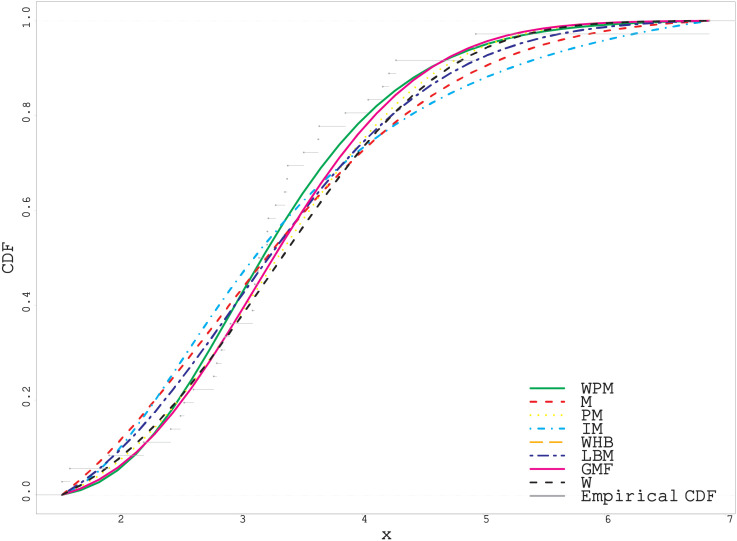
Fitted and empirical CDF plots for second real data.

**Fig 7:**
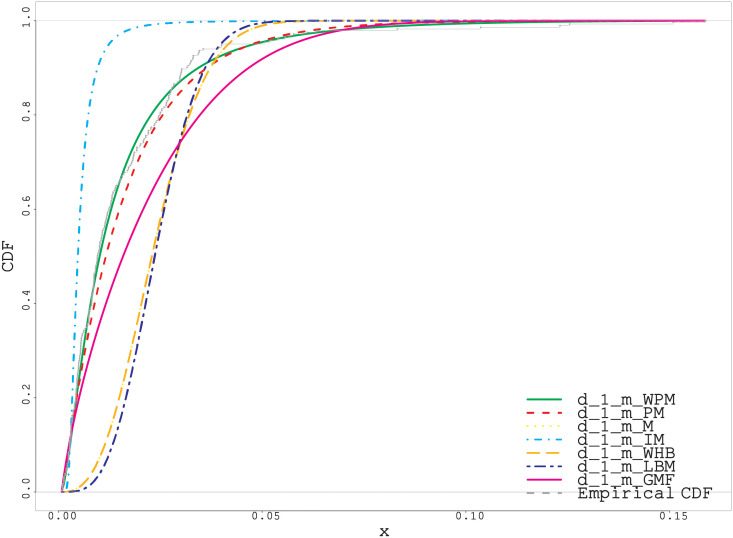
Fitted and empirical CDF plots for third real data.

As can be easily seen from the fitted and empirical CDF in Figs 5–7, the WPM distribution has the best fit for both real datasets. This is an important indicator that the WPM distribution is a good alternative to the distributions in the literature and can be used in data modeling.

## 9 Conclusion

In this work, We Propose a Novel Weighted Power Maxwell Distribution Generalization. The linear representations of the PDF and CDF, moments, moment generating function, and fuzzy reliability function have been successfully identified as a result of our investigation into its statistical features and determination of a linear representation for its PDF. For the purpose of obtaining point estimates for the unknown Weighted power Maxwell parameters *α*, *β*, and *θ*, several Bayesian and classical estimation approaches were taken into consideration. An R software was used to conduct a simulation study, which allowed for the comparison of the effectiveness of a number of different estimating strategies. To achieve this goal, the MCMC technique was used, and the results led us to the conclusion that the Bayesian approach is superior to all the traditional methods that were taken into consideration. We utilised a data collection called Covid-19 that was compiled in the United Kingdom. It was discovered that the Weighted power Maxwell distribution suited the data better than the majority of the other distributions that were being considered.

## 10 Future work

In the near future author we will make a full study on the vaccination of the patients and measure the rate of mortality after performing the vaccination an d we will try to make regression analysis to the future infection using new model.

## References

[1] Shahzad U., Ahmad I., Almanjahie I., Hanif M., and Al-Noor N. H., “L-moments and calibration based variance estimators under double stratified random sampling scheme: an application of covid-19 pandemic,” *Scientia Iranica*, 2021. doi: 10.24200/sci.2021.56853.4942

[2] Liu X., Ahmad Z., Gemeay A. M., Abdulrahman A. T., Hafez E. H., and Khalil N., “Modeling the survival times of the covid-19 patients with a new statistical model: A case study from china,” *PloS one*, vol. 16, no. 7, p. e0254999, 2021. doi: 10.1371/journal.pone.0254999 34310646 PMC8312982

[3] Nagy M., Almetwally E. M., Gemeay A. M., Mohammed H. S., Jawa T. M., Sayed-Ahmed N., and et al. “The new novel discrete distribution with application on covid-19 mortality numbers in kingdom of saudi arabia and latvia,” *Complexity*, vol. 2021, 2021. doi: 10.1155/2021/7192833

[4] Shahzad U., Ahmad I., Almanjahie I., and Al-Noor N. H., “Utilizing l-moments and calibration method to estimate the variance based on covid-19 data,” *Fresenius Environmental Bulletin*, vol. 30, no. 7A, pp. 8988–8994, 2021.

[5] Hossam E., Abdulrahman A. T., Gemeay A. M., Alshammari N., Alshawarbeh E., and Mashaqbah N. K., “A novel extension of gumbel distribution: Statistical inference with covid-19 application,” *Alexandria Engineering Journal*, vol. 61, no. 11, pp. 8823–8842, 2022. doi: 10.1016/j.aej.2022.01.071

[6] Almuqrin M. A., Gemeay A. M., Abd El-Raouf M. M., Kilai M., Aldallal R., and Hussam E., “A flexible extension of reduced kies distribution: Properties, inference, and applications in biology,” *Complexity*, vol. 2022, 2022. doi: 10.1155/2022/6078567

[7] Aldallal R., Gemeay A. M., Hussam E., and Kilai M., “Statistical modeling for covid 19 infected patient’s data in kingdom of saudi arabia,” *Plos one*, vol. 17, no. 10, p. e0276688, 2022. doi: 10.1371/journal.pone.0276688 36306316 PMC9616221

[8] Riad F. H., Alruwaili B., Gemeay A. M., and Hussam E., “Statistical modeling for covid 19 virus spread in kingdom of saudi arabia and netherlands,” *Alexandria Engineering Journal*, vol. 61, no. 12, pp. 9849–9866, 2022. doi: 10.1016/j.aej.2022.03.015

[9] Afify A. Z., Aljohani H. M., Alghamdi A. S., Gemeay A. M., and Sarg A. M., “A new two-parameter burr-hatke distribution: Properties and bayesian and non-bayesian inference with applications,” *Journal of Mathematics*, vol. 2021, 2021. doi: 10.1155/2021/1061083

[10] Maxwell J. C., “On the dynamical theory of gases,” *Philosophical transactions of the Royal Society of London*, vol. 157, pp. 49–88, 1867. doi: 10.1098/rstl.1867.0004

[11] Dey S. and Maiti S. S., “Bayesian estimation of the parameter of maxwell distribution under di erent loss functions,” *Journal of Statistical Theory and Practice*, vol. 4, no. 2, pp. 279–287, 2010. doi: 10.1080/15598608.2010.10411986

[12] Chaturvedi A. and Rani U., “Classical and bayesian reliability estimation of the generalized maxwell failure distribution,” *Journal of Statistical Research*, vol. 32, no. 1, pp. 113–120, 1998.

[13] Rao C. R. and Gupta S. D., *Selected papers of CR Rao*, vol. 5. Taylor & Francis, 1989.

[14] Hassan A. S., Almetwally E. M., Khaleel M. A., and Nagy H. F., “Weighted power lomax distribution and its length biased version: Properties and estimation based on censored samples,” *Pakistan Journal of Statistics and Operation Research*, vol. 17, no. 2, pp. 343–356, 2021. doi: 10.18187/pjsor.v17i2.3360

[15] Kilany N. M., “Weighted lomax distribution,” *SpringerPlus*, vol. 5, no. 1, pp. 1–18, 2016. doi: 10.1186/s40064-016-3489-2 27822438 PMC5078137

[16] Gupta R. C. and Tripathti R. C., “Weighted bivariate logarithmic series distributions,” *Communications in Statistics-Theory and Methods*, vol. 25, no. 5, pp. 1099–1117, 1996. doi: 10.1080/03610929608831751

[17] A. S. Yadav, H. S. Bakouch, S. K. Singh, and U. Singh, “Power maxwell distribution: Statistical properties, estimation and application,” *arXiv preprint arXiv:1807.01200*, 2018.

[18] Chen G., Pham T. T., and Boustany N. M., “Introduction to fuzzy sets, fuzzy logic, and fuzzy control systems,” *Appl. Mech. Rev*., vol. 54, no. 6, pp. B102–B103, 2001. doi: 10.1115/1.1421114

[19] J. Galambos, “The asymptotic theory of extreme order statistics,” *R.E. Krieger Pub. Co*., 1987.

[20] Arcagni A. and Porro F., “The graphical representation of inequality,” *Revista Colombiana de estadistica*, vol. 37, no. 2, pp. 419–437, 2014. doi: 10.15446/rce.v37n2spe.47947

[21] Morales D., Pardo L., and Vajda I., “Some new statistics for testing hypotheses in parametric models,” *J. Multivar. Anal*., vol. 62, no. 1, pp. 137–168, 1997. doi: 10.1006/jmva.1997.1680

[22] Kurths J., Voss A., Saparin P., Witt A., Kleiner H. J., and Wessel N., “Quantitative analysis of heart rate variability,” *Chaos: Interdiscip. J. Nonlinear Sci*., vol. 5, no. 1, pp. 88–94, 1995. doi: 10.1063/1.166090 12780160

[23] Song K. S., “Rényi information, loglikelihood and an intrinsic distribution measure,” *J. Stat. Plan. Inference*, vol. 93, no. 1-2, pp. 51–69, 2001. doi: 10.1016/S0378-3758(00)00169-5

[24] Lawless J. F., *Statistical models and methods for lifetime data*. John Wiley & Sons, 2011.

[25] Anatolyev S., and Kosenok G., “An alternative to maximum likelihood based on spacings,” *Econometric Theory*, vol. 21, no. 2, pp. 472–476, 2005. doi: 10.1017/S0266466605050255

[26] Greene W. H., *Econometric analysis*. Pearson Education India, 2003.

[27] El-Sherpieny E. A., Muhammed H. Z., and Almetwally E. M., “Bivariate weibull-g family based on copula function: properties, bayesian and non-bayesian estimation and applications,” *Statistics*, *Optimization & Information Computing*, 2021. doi: 10.19139/soic-2310-5070-1129

[28] Calabria R. and Pulcini G. s., “An engineering approach to bayes estimation for the weibull distribution,” *Microelectronics Reliability*, vol. 34, no. 5, pp. 789–802, 1994. doi: 10.1016/0026-2714(94)90004-3

[29] Abu El Azm W. S., Almetwally E. M., Naji S. A., El-Bagoury A. H., Alharbi R., and Abo-Kasem O. E., “A new transmuted generalized lomax distribution: Properties and applications to covid-19 data,” *Computational Intelligence and Neuroscience*, 2021. doi: 10.1155/2021/5918511 PMC8497124 34630548

[30] Almetwally E. M., Alharbi R., Alnagar D., and Hafez E. H., “A new inverted topp-leone distribution: applications to the covid-19 mortality rate in two different countries,” *Axioms*, vol. 10, no. 1, p. 25, 2021. doi: 10.3390/axioms10010025

[31] Omar M. H., Arafat S. Y., Hossain M., and Riaz M., “Inverse maxwell distribution and statistical process control: An e cient approach for monitoring positively skewed process,” *Symmetry*, vol. 13, no. 2, p. 189, 2021. doi: 10.3390/sym13020189

[32] Dar A. A., Ahmed A., and Reshi J., “Characterization and estimation of weighted maxwell-boltzmann distribution,” *Applied Mathematics & Information Sciences*, vol. 12, no. 1, pp. 193–202, 2018. doi: 10.18576/amis/120119

[33] Saghir A., Khadim A., and Lin Z., “The maxwell length-biased distribution: Properties and estimation,” *Journal of Statistical theory and Practice*, vol. 11, no. 1, pp. 26–40, 2017. doi: 10.1080/15598608.2016.1246266

